# Cloning of wheat *keto-acyl thiolase 2B* reveals a role of jasmonic acid in grain weight determination

**DOI:** 10.1038/s41467-020-20133-z

**Published:** 2020-12-08

**Authors:** Yun Chen, Yan Yan, Tian-Tian Wu, Guo-Liang Zhang, Huanran Yin, Wei Chen, Shuangshuang Wang, Fang Chang, Jin-Ying Gou

**Affiliations:** 1 State Key Laboratory of Genetic Engineering, MOE Key Laboratory for Biodiversity Science and Ecological Engineering, MOE Engineering Research Center of Gene Technology, Institute of Plant Biology, School of Life Sciences, Fudan University, Shanghai, 200438 China; 2 Institute of Metabolism and Integrative Biology, Fudan University, Shanghai, 200438 China; 3 National Key Laboratory of Crop Genetic Improvement and National Center of Plant Gene Research (Wuhan), Huazhong Agricultural University, Wuhan, 430070 China

**Keywords:** Agricultural genetics, Agricultural genetics, Jasmonic acid

## Abstract

Grain weight (GW) is one of the component traits of wheat yield. Existing reports have shown that multiple phytohormones are involved in the regulation of GW in different crops. However, the potential role of jasmonic acid (JA) remains unclear. Here, we report that *triticale grain weight 1* (*tgw1*) mutant, with marked reductions in both GW and JA content, is caused by a premature stop mutation in *keto-acyl thiolase 2B* (*KAT-2B*) involved in β-oxidation during JA synthesis. *KAT-2B* overexpression increases GW in wild type and boosts yield. Additionally, *KAT-2B* compliments the grain defect in *tgw1* and rescues the lethal phenotype of the *Arabidopsis kat2* mutant in a sucrose-free medium. Despite the suppression of JA synthesis in *tgw1* mutant, ABA synthesis is upregulated, which is accompanied by enhanced expression of *SAG3* and reduction of chlorophyll content in leaves. Together, these results demonstrate a role of the JA synthetic gene *KAT-2B* in controlling GW and its potential application value for wheat improvement.

## Abstract (web summary)

It has been reported that several phytohormones are involved in the regulation of grain weight, but the role of jasmonic acids (JA) remains elusive. Here, via bulked segregant analysis (BSA)-based cloning, the authors show keto-acyl thiolase 2B involved in β-oxidation during JA synthesis is a positive regulator of wheat grain weight.

## Introduction

As significant energy and protein resources for human beings, wheat is needed to increase another 50% from the current amount to feed the ever-growing world population by 2034 (International Wheat Yield Partnership). Wheat yield is mainly determined by three major dimensions: number of spikes per unit area, number of grains per spike, and grain weight (GW). The first two dimensions determine the grain number (GN) per unit area of land, a dominant component for yield in environments with favorable growth conditions^1^. However, there is a trade-off between GN and GW^2^. Nevertheless, an increase of GW occurred in emmer wheat during domestication^3^. Therefore, GW enhancement is a feasible strategy to improve cereal yield depending on the environment^1,4,5^.

Grain size (GS) positively correlates with GW and has effectively promoted grain yield in rice^6^. Major quantitative trait loci and regulatory genes were cloned, which control the size and shape of rice grains through three major pathways, i.e., degradation of protein through the ubiquitin-proteasome process, signaling mediated by G protein/protein kinase, and plant phytohormone signal transductions mediated by auxin, cytokinin, and brassinosteroids^7–16^. Nevertheless, little studies reported the regulation of GS and GW by jasmonic acid (JA).

JA, as an essential plant hormone, fulfills critical roles in plant defense and development^17^. For example, JA confers pleiotropic effects on pollen grain and embryo developments^18^. In rice, downregulation of endogenous JA level by a 12-bp insertion in an enoyl-CoA hydrolase gene (*NOG1*) increased GN and grain yield^19^. A rice plastid-targeted lipase, EG2/OsJAZ1, has a critical regulatory role in spikelet development^20^. A wheat allene oxide cyclase (AOC) catalyzes a crucial step in the JA biosynthesis pathway and enhances salt tolerance in transgenic plants^21^. Besides, AOC is strongly induced in *Leymus mollis*, a wild relative of wheat grown along sea coasts, and can be introduced into wheat to enhance salt tolerance^22^. Beta-oxidation reaction catalyzes the chain shorting step in JA synthesis, fatty-acid degradation and conversion of indole-3-butyric acid (IBA) to indoleacetic acid (IAA). Ketoacyl thiolase (KAT) is an essential enzyme in the beta-oxidation reaction. The potential effect of JA on wheat GS and final yield is still unknown.

Here, we report the isolation of a *triticale grain weight 1* (*tgw1*) mutant from a tetraploid wheat EMS mutant library. We show that *tgw1* produces smaller grains and accumulates less JA. This mutant contains a premature stop mutation in a 3-*ketoacyl-CoA thiolase* (*KAT-2B*) gene functioning in JA synthesis. The biological function of *KAT-2B* is confirmed by overexpression in wild type (WT), *tgw1*, and *Arabidopsis kat2* mutant. We show that *KAT-2B* overexpressors increase thousand kernel weight (TKW) and grain yield in field trials. These findings indicate that *KAT-2B* is valuable in dissecting the regulations of GS, GW, and GN–GW balance, as well as investigating JA’s biological functions in wheat grain development.

## Results

### Phenotypic characterization of *triticale grain weight 1*

**Fig. 1:**
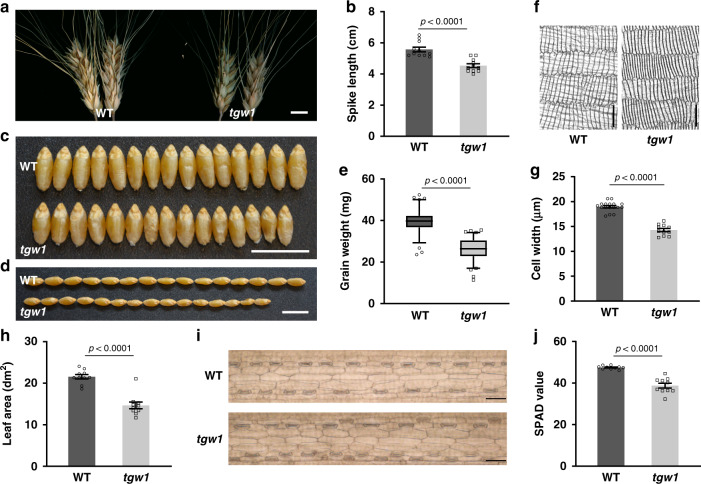
Identification and characterization of *tgw1*. **a** Representative images of main tiller spikes from WT or *tgw1*. Scale bar = 1 cm. **b** Average length of main spikes from WT and *tgw1*. *n* = 11. **c–d** Comparison of width and length of mature grains from WT or *tgw1*. Scale bar = 1 cm. **e** Average grain weight in WT and *tgw1*. *n* = 80. **f** Representative images of grain endodermal cells in WT and *tgw1*. Scale bar = 100 μm. **g** Average width of grain endodermal cell in WT (*n* = 17) and *tgw1* (*n* = 11). **h** The average area of flag leaves in WT and *tgw1* mutant. *n* = 10. **i** Leaf epidermal cells in WT or *tgw1* mutant. Scale bar = 100 μm. **j** Average chlorophyll units in flag leaves from WT and *tgw1* mutant. *n* = 10. Data in **e** are represented as boxplots. The boxplots indicate median (middle line), 25^th^, 75^th^ percentile (box), and 5^th^ and 95^th^ percentile (whiskers) as well as outliers (single points). Data in **b**, **e**, **g**, **h**, and **j** are represented as mean ± SEM, and *p* values are indicated by two-tailed unpaired *t* test. Results in **f** and **i** are representative of three independent samples. Source data are provided as a Source Data file.

We developed an EMS mutant library using tetraploid spring wheat (Kronos, WT hereafter) and screened for *triticale grain weight* (*tgw*) mutants with significant changes in TKW. *tgw1* had shorter spikes than WT (Fig. 1a, b). At the full maturation stage, *tgw1* grains exhibited evident reductions in length and width compared with WT (Fig. 1c, d). The average GW of *tgw1* was 26 ± 0.6 mg, which was significantly lower than WT control (39 ± 0.6 mg) in a growth chamber (Fig. 1e). Microscopical examination revealed that the endodermal cells of *tgw1* were significantly narrower than those of the WT line (Fig. 1f, g).

Compared with WT, *tgw1* showed a significant reduction in the leaf area (Fig. 1h). However, there was no difference in the average area of cells in the leaf (Fig. 1i, Supplementary Fig. 1), suggesting a potential reduction in cell numbers instead. Furthermore, there is a significant reduction in the mutant’s chlorophyll contents than WT (Fig. 1j). Therefore, both changes in the leaf area and chlorophyll content were unfavorable for the pool’s energy source, contributing to the GW change.

### Molecular cloning of *TGW1*

**Fig. 2:**
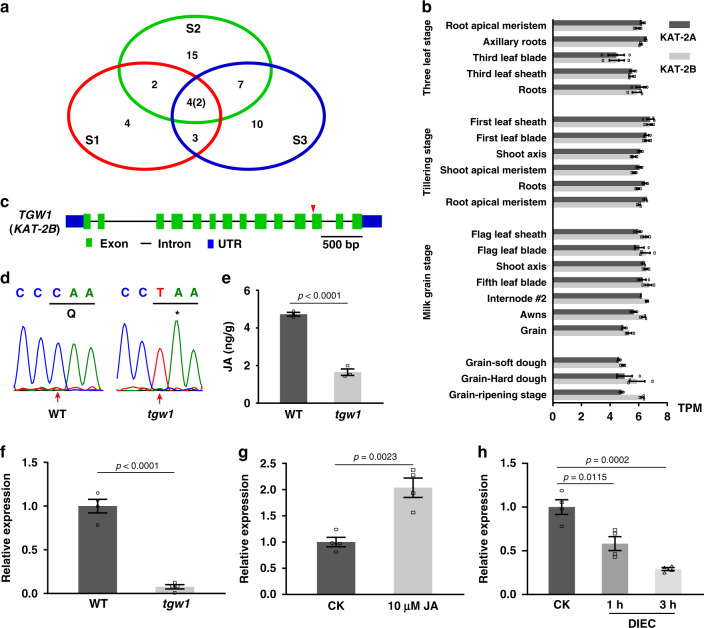
Molecular cloning of *TGW1*. **a** Venn diagram to show distributions of homozygous SNPs in three groups of homozygous BC_1_F_2_ mutants. S1 to S3 represented three groups of homozygous BC_1_F_2_ mutants. Each number represents numbers of homozygous SNPs. Only two nonsynonymous homozygous SNPs presented in three groups. **b** The expression of *KAT-2B* (TraesCS6B01G432600) and *KAT-2A* (TraesCS6A01G392400) in different tissues at various developmental stages. *n* = 3 independent replicates. **c** Genomic structure of *TGW1* (*KAT-2B*) gene. The red triangle points to the position of the point mutation in *tgw1*. **d** Verification of the SNP in the *KAT-2B* (*TGW1*) gene in WT and *tgw1* by sequencing. **e** Contents of JA in leaves of WT and *tgw1*. *n* = 3. **f** Relative expression level of *Cell Number Regulator 6* (*CNR6*) in WT and *tgw1* mutant. *n* = 4. **g** The expression level of *CNR6* upon 10 μM JA treatments in WT. *n* = 4. **h** The expression level of *CNR6* upon 200 μM JA inhibitor DIEC treatments in WT. *n* = 4. Data are represented as mean ± SEM in **b**, and **e**–**h**. *p* values are indicated by two-tailed unpaired *t* test in **e**–**h**. Source data underlying Figs. 2a, b, e–h are provided as a Source Data file.

Bulked segregant analysis (BSA) identified potential correlations between phenotype and genotype in BC_1_F_2_ generation. A total of 40 Giga data were sequenced from three samples (S1, S2, S3), each containing eight individual homozygous BC_1_F_2_ plants. Only two nonsynonymous homozygous SNPs, located in *TraesCS6B02G432600* and *TraesCS6B02G414800*, respectively, were detected in all three samples (Fig. 2a).

To investigate which of the two genes may be the candidate *TGW1* gene, we first checked their expression levels in wheat according to the digital Northern data extracted from public microarray results (GSE12508 in NCBI). The expression of *TraesCS6B02G414800* was low in most tissues (Supplementary Fig. 2). The other gene, *TraesCS6B02G432600*, annotated to encode KAT-2, had a signal of over 20 fold higher than that of *TraesCS6B02G414800* in all the analyzed tissues (Supplementary Fig. 2).

Based on 71 RNA-seq studies in different tissues at various developmental stages (http://bar.utoronto.ca/efp_wheat/cgi-bin/efpWeb.cgi), the expression of *KAT-2B* (TraesCS6B01G432600) was higher than *KAT-2A* (TraesCS6A01G392400) (Fig. 2b, Supplementary Data 1). There was a CAA to TAA mutation in the 12^th^ exon of *KAT-2B* in *tgw1* (Fig. 2c), which introduced a premature stop codon (Q364*) at a position with 92 amino acids to the C terminal end of KAT-2 (Fig. 2d). Based on the KAT-2′s structure, two out of the three active sites (H-407 and N-447) are critical for KAT-2 activity located after Q364^23^. Hence, the Q364* mutation should have an inhibitory effect on the biochemical activity of KAT-2. In *Arabidopsis*, *kat2* mutant also showed reduced reproductive success, with substantially seed less yield and single seed weight^24,25^.

KAT2 catalyzes an essential step in β-oxidation reaction to reduce carbon chain length and produce acetyl CoA during fatty-acid degradation and the biosynthesis of two crucial plant hormones, IAA and JA^24,25^. To explore potential metabolite changes between WT and *tgw1*, we quantified contents of fatty acids by GC/MS, IAA by LC/MS, and JA by enzyme-linked immunosorbent assay (ELISA). No significant differences showed up in free fatty acids profiles or IAA contents between *tgw1* and WT (Supplementary Fig. 3). However, the JA content in the *tgw1* mutant was 50% less than that of WT, a significant change (Fig. 2e). The level of oxidized lipid was also lower in *tgw1* than in WT (Supplementary Fig. 4).

In the *tgw1* mutant, a gene annotated as a cell number regulator (*CNR6*) significantly decreased at the expression level (Fig. 2f), which correlated to the cell number changes in the *tgw1* mutant. Upon JA treatment, the expression of *CNR6* significantly increased in seedling leaves (Fig. 2g). On the contrary, the JA inhibitor application, sodium diethyldithiocarbamate (DIEC), suppressed the expression of *CNR6* in a time-dependent manner (Fig. 2h). Therefore, both exogenous JA and inhibitor treatment suggested JA’s role in regulating *CNR6* expression, as shown in the *tgw1* mutant.

Moreover, the *tgw1* mutant produced smaller anthers with fewer pollen grains (Supplementary Fig. 5a), many of which were defective (Supplementary Fig. 5b–d). Quantitative analysis showed that the defective pollen grains of *tgw1* (11.8%) was significantly higher than that of WT control (4%) (Supplementary Fig. 5e). The above changes were consistent with early reports that JA played an essential role in the development of pollens in model plants^18,26^. These results suggested that wheat *KAT-2B* is very likely involved in the biosynthesis of JA.

### Validation that *KAT-2B* is *TGW1*

**Fig. 3:**
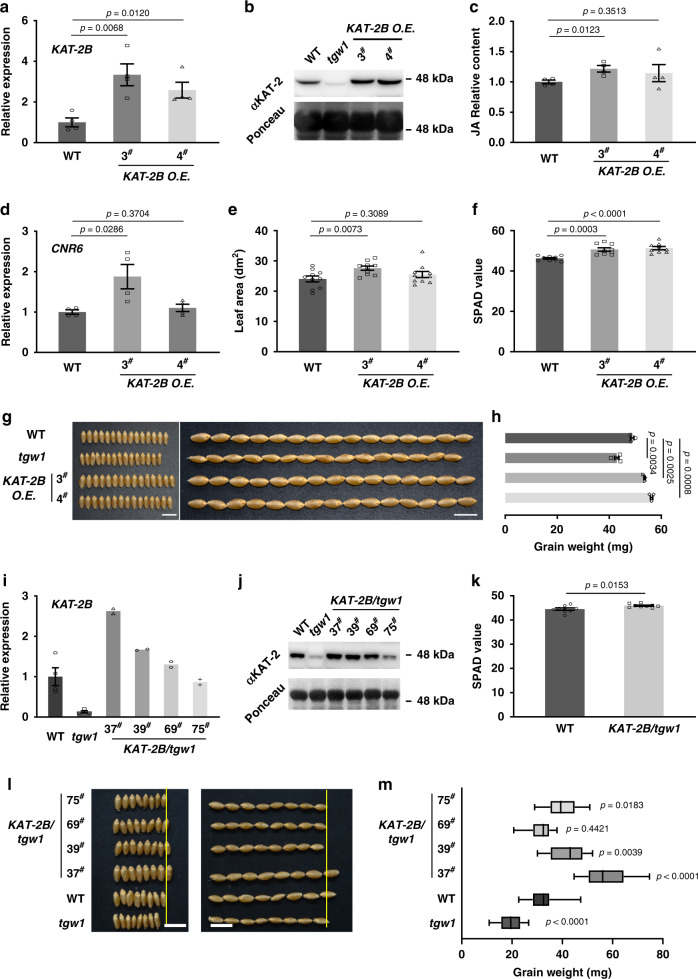
The biological function of *KAT-2B* in transgenic plants. **a** Comparison of *KAT-2B* expression level by qRT-PCR in WT, and transgenic wheat. *n* = 4. **b** Western blot analysis of KAT-2. Ponceau staining of RuBisCO serves as a loading control. **c** Relative quantification of JA in WT and transgenic wheat. *n* = 4. WT was set to 1. **d** Expression level of *CNR6* in transgenic wheat. *n* = 4. **e**–**f** Comparison of average flag leaf areas **e** and chlorophyll content **f** in WT and transgenic wheat. *n* = 10 **e**. *n* = 9 **f**. **g** Comparison of grains from in WT, *tgw1* mutant, and *KAT-2B* transgenic lines. Scale bar = 1 cm. **h** Average weight of grains from WT (*n* = 3), *tgw1* mutant (*n* = 4), and transgenic lines (*n* = 3). **i**–**j** Comparison of *KAT-2B* expression (i) and protein (j) levels in *tgw1* mutant (*n* = 4), WT (*n* = 4), and complementation lines. **k** Comparison chlorophyll contents in *KAT-2B*/*tgw1* and *tgw1* mutant lines. *n* = 9. **l** Comparison of grains from in *KAT-2B*/*tgw1*, WT, and *tgw1* mutant lines. Scale bar = 1 cm. **m** Comparison of average grain weight in *KAT-2B*/*tgw1*, WT, and *tgw1* mutant. *n* = 12. Results in **b** and **j** are representative of two independent experiments. In **m** data are represented as boxplots. The boxplots indicate median (middle line), 25^th^, 75^th^ percentile (box) and 5^th^ and 95^th^ percentile (whiskers). Data from complementation lines in **i** are represented as mean from two technical replicates. Data in **a**, **c**–**f**, **h**, **i** (WT, *tgw1*), **k** and **m** are represented as mean ± SEM from independent biological replicates, and *p* values are indicated by two-tailed unpaired *t* test. Source data underlying Fig. 3a–k, m are provided as a Source Data file.

First, we introduced a *KAT-2B* gene (*Pro*_*Ubi*_: *KAT-2B*-*Flag*) into the WT wheat line by genetic transformation. In a quantitative analysis, the *KAT-2B* expression level was higher in the transgenic plants than WT (Fig. 3a). At the protein level, KAT-2 protein showed an apparent reduction in *tgw1* but accumulated at a higher level in the transgenic overexpression lines (Fig. 3b). *KAT-2A* could very likely encode the residual KAT-2 in the *tgw1* mutant, expressed at a relatively lower level (Fig. 2b). In seedlings, JA content was significantly higher in the transgenic line *KAT-2B O.E*. 3^#^ (Fig. 3c). In transgenic line 4 with no change in seedlings, the JA content significantly increased in leaves treated upon pathogen treatment (Supplementary Fig. 6).

Next, the expression level of *CNR6* in the transgenic lines was evaluated by real-time quantitative PCR (qRT-PCR). In *KAT-2B O.E*. 3^#^, *CNR6* expressed at a significantly higher level than WT (Fig. 3d). Consistently, the flag leaf area increased by 14.5% and 9.2% in *KAT-2B O.E*. 3^#^ and *KAT-2B O.E*. 4^#^, respectively (Fig. 3e). In the chlorophyll content, both lines showed increases of 9.5–11.1%, which were very significant compared with WT (Fig. 3f). Overall, both leaf area and chlorophyll content indicated that the photosynthesis capacity should increase in the *KAT-2B* transgenic lines. As a result, the transgenic grains displayed a definite increase in width compared with WT but had no apparent difference in length (Fig. 3g). Contrary to *tgw1*, *KAT-2B* transgenic lines had an average GW of 45 mg, which was significantly higher than WT (Fig. 3h).

The agricultural traits of *KAT-2B* transgenic, *tgw1*, and WT were evaluated in the field in June 2020. Consistent with the leaf defects in *tgw1*, grain number per spike (GNS) and grain weight per spike (GWS) in *tgw1* reduced by 7.3% and 18.3%, respectively, significantly lower than that of WT (Table 1). However, GNS in the *KAT-2B O.E*. transgenic line main spikes was reduced by 2.4–9.3% (Table 1), reflecting a GN–GW trade-off reported previously^2^. In the transgenic lines, the grain yield per plant increased by 18% to 28% than that of WT. The transgenic event of *KAT-2B* in wheat led to an over 15% increase in the final yield per unit area in the two transgenic lines (Table 1). These data were consistent with the field trial in June 2019 (Supplementary Table 1). These results agreed with the leaf area and chlorophyll contents changes, as the source limitation to GW in modern wheat cultivars^26^.

**Table 1:** Agricultural traits of WT, *tgw1*, and *KAT-2B* overexpression lines in the field in June 2020.

	WT	*tgw1*	*KAT-2B O.E*. 3^#^	*KAT-2B O.E*. 4^#^
TN	3.59 ± 0.78 ^*n* = 63^	3.55 ± 0.59 ^*p* = 0.7791, *n* = 42^	5 ± 1.34 ^*p* < 0.0001, *n* = 20^	4.77 ± 1.02 ^*p* < 0.0001, *n* = 22^
SPS	17.39 ± 1.03 ^*n* = 88^	16.07 ± 1.03 ^*p* < 0.0001, *n* = 63^	17.13 ± 1.6 ^*p* = 0.2705, *n* = 40^	17.67 ± 1.11 ^*p* = 0.1703, *n* = 39^
SPL (cm)	7.21 ± 0.54 ^*n* = 88^	6.29 ± 0.52 ^*p* < 0.0001, *n* = 61^	7.48 ± 0.55 ^*p* = 0.0106, *n* = 40^	7.41 ± 0.48 ^*p* = 0.0509, *n* = 40^
GNS	44.78 ± 5.76 ^*n* = 87^	41.49 ± 6.66 ^*p* = 0.0022, *n* = 61^	43.7 ± 4.54 ^*p* = 0.2971, *n* = 40^	40.62 ± 4.35 ^*p* = 0.0001, *n* = 37^
GWS (g)	2.18 ± 0.33 ^*n* = 53^	1.78 ± 0.34 ^*p* < 0.0001, *n* = 63^	2.31 ± 0.24 ^*p* = 0.0385, *n* = 38^	2.16 ± 0.21 ^*p* = 0.7565, *n* = 32^
TKW (g)	49.37 ± 0.97 ^*n* = 7^	45.9 ± 0.34 ^*p* < 0.0001, *n* = 5^	51.43 ± 0.39 ^*p* = 0.0012, *n* = 5^	51.35 ± 0.73 ^*p* = 0.0033, *n* = 5^
GYP (g)	7.49 ± 2.35 ^*n* = 62^	5.51 ± 1.28 ^*p* < 0.0001, *n* = 42^	9.64 ± 2.39 ^*p* = 0.004, *n* = 20^	8.9 ± 2.58 ^*p* = 0.0250, *n* = 22^
GY (ton)	3.96 ± 0.12 ^*n* = 4^	3.52 ± 0.09 ^*p* = 0.0011, *n* = 4^	5.26 ± 0.36 ^*n* = 2^	4.57 ± 0.03 ^*n* = 2^

*TN* Tiller number at harvest, *SPS* total spikelet number per spike, *SPL* spike length (cm), *GNS* grain number per spike, *GWS* grain weight per spike, *TKW* thousand kernel weight, *GYP* grain yield per plant, *GY* grain yield per hectare. Data are represented as mean ± SD, and *p* values are indicated by two-tailed unpaired *t* test. Please refer to Supplementary Table 1 for June 2019 field trial data. Source data are provided as a Source Data file.

To confirm that KAT-2B^Q364*^ is *tgw1*, we crossed *KAT-2B* transgenic plant with *tgw1* to overexpress wild-type *KAT-2B* into *tgw1* (*KAT-2B*/*tgw1*). All four complementation lines accumulated variable KAT-2B (Fig. 3i, j). The chlorophyll content was also restored in the complementation lines (Fig. 3k). The grains displayed apparent increases in width compared with *tgw1* (Fig. 3l). Based on GW, complete complementation of the *tgw1* phenotype was observed in all four lines, demonstrating that *KAT-2B* transgene successfully rescued the GW defect in *tgw1* (Fig. 3m). Thus, both BSA (Fig. 2a) and complementation results validated *KAT-2B* as *TGW1*.

### Molecular characterization of *KAT-2B*

**Fig. 4:**
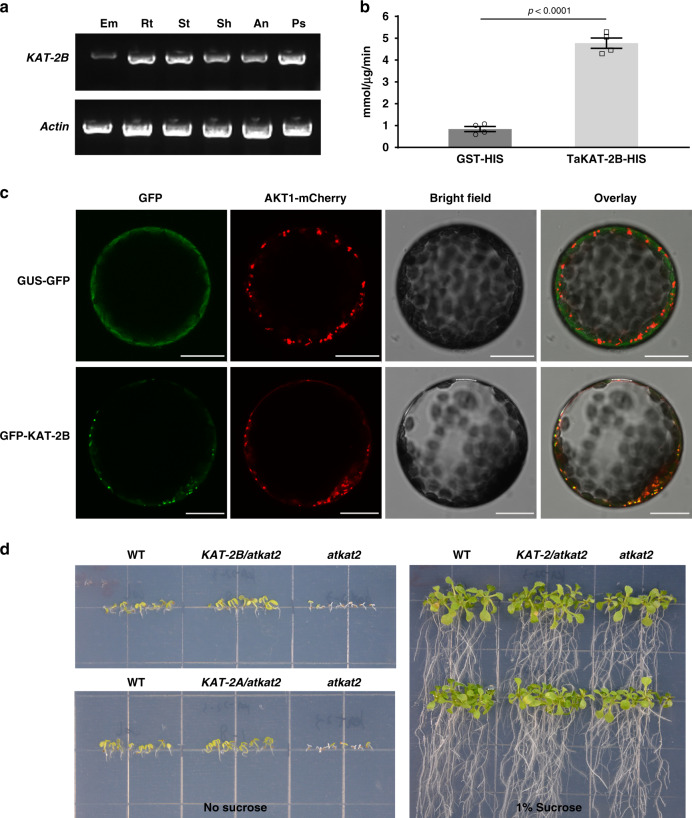
*KAT-2B* is an active thiolase in the peroxisome. **a** RT-PCR analysis of *KAT-2B* in six tissues of WT. **b** Specific biochemical activity of recombinant KAT-2B. *n* = 4. Data are represented as mean ± SEM, and *p* values are indicated by two-tailed unpaired *t* test. **c** Subcellular localization of GFP-KAT-2B fusion protein in protoplast. Images are representative of three independent experiments. GUS-GFP stands for a soluble protein. AKT1-mCherry is a peroxisome marker. Scale bar = 25 μm. **d** Rescue of the *Arabidopsis kat2* mutant in a sucrose-free medium by wheat *KAT-2* transgene. Note that *Arabidopsis kat2* mutant is lethal in a sucrose-free medium. Each grid represents 1 cm^2^ on the plate. Source data underlying Fig. 4a–c are provided as a Source Data file.

After validating the function of *KAT-2B* by genetics, we then investigated more basic molecular characteristics of *KAT-2B*. As shown by RT-PCR, *KAT-2B* highly expressed in most tissues except embryo (Fig. 4a), in agreement with the public microarray data (Supplementary Data 1).

To examine the biochemical activity of KAT-2B, we purified KAT-2B recombinant protein. Thiolase activity was readily detected for recombinant KAT-2B using acetoacetyl CoA and CoA as substrates, representing a typical carbon chain shortening step in β-oxidation (Fig. 4b). Moreover, the specific activity of KAT-2B was activated by some monovalent cations (K^+^ and Na^+^) but inhibited by certain divalent cations (e.g., Mg^2+^) in vitro (Supplementary Fig. 7a). The recombinant KAT-2B protein preferred alkaline conditions (pH, ranging from 7.5 to 9) (Supplementary Fig. 7b).

To further test the involvement of *KAT-2B* in the β-oxidation process, we fused *KAT-2B* with *GFP* in a transgenic vector (*Pro*_*35S*_: *GFP-KAT-2B*) and transiently co-expressed it with a peroxisome marker, AKT1-mCherry^27^, in tobacco leaves. Under a confocal microscope, GFP-KAT-2B co-localized with AKT1-mCherry in leaf epidermal cells (Supplementary Fig. 8). In the protoplast prepared from mesophyll cells, GFP-KAT-2B fusion protein also co-localized with AKT1-mCherry (Fig. 4c). These data suggested that KAT-2B performed its biological function in the peroxisome, the organelle where the β-oxidation reaction occurs.

The role of wheat *KAT-2* in β-oxidation was also validated by complementation of *Arabidopsis kat2* mutant, which cannot convert endogenous lipids into acetyl CoA to produce energy; therefore is lethal in ½ MS medium without sucrose as the solo exogenous energy resource^28^. *KAT-2* homeologs from the A and B genomes of wheat were transformed into *Arabidopsis kat2* homozygous mutant (Supplementary Fig. 9). As expected, the resultant *KAT-2 Arabidopsis* transgenic lines grew well on the sucrose-free medium (Fig. 4d), indicating that wheat *KAT-2* in both A and B genomes could entirely rescue the β-oxidation defect in *Arabidopsis kat2* mutant.

### Plant hormone biosynthesis changes in *tgw1*

**Fig. 5:**
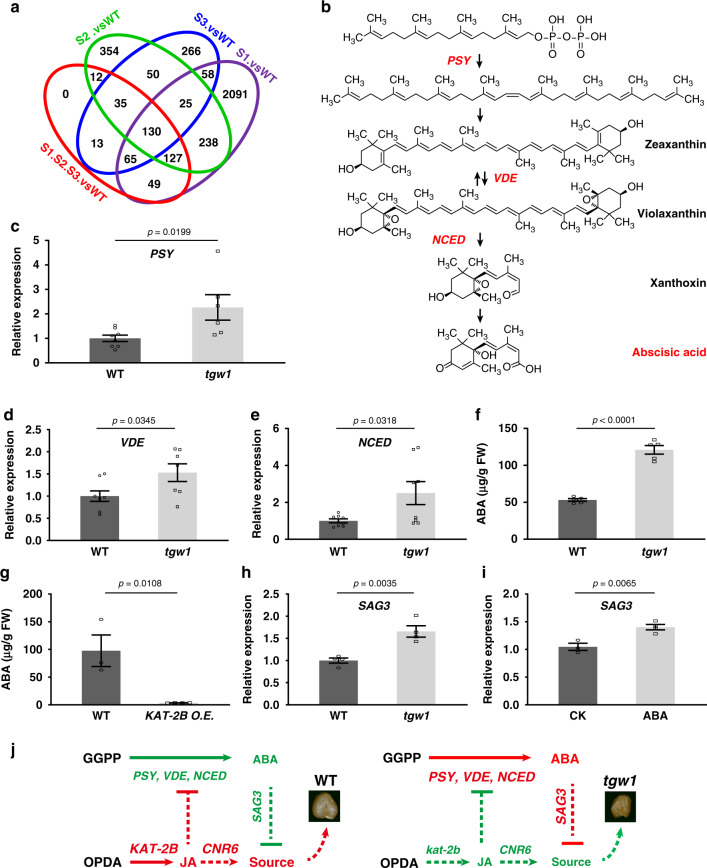
Regulation of ABA synthesis in *tgw1*. **a** Venn diagram representation of differential expression genes between WT and the *tgw1* backcrossed mutant. **b** Schematic representation of the ABA synthesis pathway. *PSY*, *VDE*, and *NCED* were upregulated in *tgw1* mutant. **c**–**e** Relative expression level of *PSY*, *VDE*, and *NCED* in WT and *tgw1* mutant from at least four independent biological samples. **f** Quantification of ABA in WT and *tgw1* mutant. *n* = 5. **g** Quantification of ABA in WT (*n* = 3) and transgenic plants (*n* = 4). **h** Relative expression level of *senescence-associated gene 3* (*SAG3*) in WT and *tgw1* mutant. *n* = 4. **i** Expression change of *SAG3* upon 80 μM ABA treatment. *n* = 4. **j** A predicted working model for GW regulation in WT or *tgw1* mutant. Red, upregulated. Green, downregulated. WT and *tgw1* grains were cross-dissected to show grain width. Data in **c**–**i** are represented as mean ± SEM, and *p* values are indicated by two-tailed unpaired *t* test. Source data underlying Fig. 5a and c–i are provided as a Source Data file.

The expression change of *CNR6* could partially contribute to the leaf area changes in the *tgw1* mutant line. Nevertheless, there is no clue for the changes in the chlorophyll contents in the *tgw1* mutant. To investigate processes involved in the regulation of GW and other traits, we compared the transcriptome of the three groups of plants in *tgw1* BC_1_F_2_ generation with WT (Fig. 5a, Supplementary Data 2).

Interestingly, the expression levels of several genes in phytohormone metabolism altered in *tgw1*, including the suppression of 14 genes in JA synthesis and one gene in gibberellic acid (GA) biosynthesis (Table 2). The former was consistent with the change of oxidized lipids in *tgw1* (Supplementary Fig. 4). ABA synthesis pathway genes, however, were upregulated, including *phytoene synthase* (*PSY*, a committed step to flux GGPP into carotenoid synthesis), *violaxanthin de-epoxidase* (*VDE*), and 9-cis-epoxycarotenoid dioxygenase (*NCED*, a crucial step for ABA synthesis from carotenoid) (Fig. 5b). qRT-PCR experiments further confirmed the up-regulation of these genes in *tgw1* (Fig. 5c–e).

**Table 2:** List of differential genes involved in plant hormone metabolism between *tgw1* and WT.

Gene-ID	log_2_(fold change)	Annotation
*JA*		
TraesCS2A02G525500.1	−1.39	Lipoxygenase
TraesCS2B02G555400.1	−1.49	Lipoxygenase
TraesCS4A02G061800.1	−1.49	Allene oxide synthase
TraesCS4A02G061900.1	−1.13	Allene oxide synthase
TraesCS4B02G037700.2	−1.11	Lipoxygenase
TraesCS4B02G037900.1	−1.67	Lipoxygenase
TraesCS4B02G237600.1	−1.21	Allene oxide synthase
TraesCS5A02G007900.1	−1.28	Lipoxygenase
TraesCS5B02G006500.1	−1.24	Lipoxygenase
TraesCS6B02G160400.1	−1.64	Lipoxygenase
TraesCS6B02G432600.1	−4.07	3-ketoacyl-CoA thiolase-like
TraesCS6B02G056800.2	−1.18	Catalase
TraesCS6A02G041700.1	−1.23	Catalase
TraesCS1A02G030400.1	−2.00	Jasmonate O-methyltransferase
*ABA*		
TraesCS5A02G020900.1	1.07	Phytoene synthase, chloroplastic-like
TraesCS2B02G297800.1	1.24	Violaxanthin de-epoxidase
TraesCS5A02G374000.1	1.92	9-cis-epoxycarotenoid dioxygenase
*GA*		
TraesCS2A02G425500.1	−1.16	Kaurene synthase

All the above genes showed two-sided adjusted *p* value (FDR) ≤ 0.05 between the three groups of *tgw1* BC_1_F_2_ lines (S1 to S3) and WT.

The ABA content showed a significant increase in the *tgw1* mutant (Fig. 5f). On the contrary, the *KAT-2B* transgenic lines accumulated less ABA (Fig. 5g), suggesting a negative correlation between the biological function of KAT-2B (JA content) and ABA biosynthesis in wheat. To be consistent with the ABA content in the *tgw1* mutant, a wheat leaf senescence-associated marker gene, *SAG3*^29^, increased in a qRT-PCR assay (Fig. 5h). Exogenous ABA treatment enhanced the expression of *SAG3* in young seedlings (Fig. 5i).

The above data suggested that the JA synthesis defect heightened the expression of ABA synthesis genes and increased ABA, which enhanced the expression of *SAG3*. Early senescence marked by *SAG3* increase could be a potential link between the increased ABA and reduced chlorophyll contents in the *tgw1* mutant, as the other regulation, besides leaf area, on the source limitation for grains (Fig. 5j).

## Discussion

Through this work, we discovered a unique role of wheat *KAT-2B* in regulating GW through directly controlling de novo JA synthesis. However, the mutational effects of the wheat *KAT-2B* gene are pleiotropic, e.g., negatively affecting JA content and leaf and pollen development, and positively increasing ABA content. Therefore, the mechanism underlying the *KAT-2B* function is likely very complicated.

In our study, wheat *tgw1* led to expression changes of multiple essential synthesis genes in several plant hormones, e.g., JA, ABA, and GA (Table 2). In metabolite analyses, JA showed an apparent decrease, whereas no significant change could be detected in the contents of IAA and total free lipids between *tgw1* and the control samples analyzed. β-oxidation was reported to catalyze the conversion of IBA to IAA, which affects root hair elongation and cotyledon size^30^. In the plant, however, IAA was mainly synthesized from l-tryptophan via indole-3-pyruvate by the TAA and YUC families in *Arabidopsis*^31^. Our data here agreed with the latter report that β-oxidation catalyzed by KAT-2B might not play a significant role in IAA synthesis in wheat.

Oxidized lipids, including JA, are reported at low levels in plants in earlier studies. In *Arabidopsis*, oxidized lipids composition was changed induced upon treatment at 4 °C or pathogen inoculation, but the highest amount upon treatment (~4.3 nmol per mg of dry mass) was still much lower than those of normal-chain phospholipids and galactolipids (~259 nmol per mg of dry mass)^32^. In wheat, oxidized lipids were reported to increase upon high-temperature treatment in the leaves of a susceptible cultivar^33^. However, the contents of ox-lipids ranged from 0.0005 to 0.015 mass spectral signal per mg dry mass, which was relatively low compared to the total lipids (50–200)^33^. Therefore, the change in JA content could only have a minor effect on the total content of lipids, which was an under-detectable level in our system.

In most plants, phytohormones are essential regulators of plant leaf senescence, and many of these altered senescence phenotypes occur as a result of altered hormone signaling^34^. In *Arabidopsis*, a JA-inducible gene, *Dof2.1* (DNA binding-with-one-finger), acted as an enhancer of JA-induced leaf senescence through the MYC2-Dof2.1-MYC2 feed-forward transcriptional loop. In addition to the decrease of JA, the up-regulation of ABA could also be important for the grain defect in *tgw1*. The *Arabidopsis kat2* mutant showed an increase in ABA response^35^. In rice, an NAM/ATAF½/CUC2 (NAC) transcription factor ONAC054 bound the promoter region and played a crucial role in the regulation of ABA signaling and senescence-related genes, e.g., *OsABI5* and *NYC1*^36^. A MYB-related transcription factor *Oryza sativa* RADIALIS-LIKE3 (OsRL3), induced by ABA, promoted the expression levels of chlorophyll-degradation and senescence-associated genes^37^. In maize, ABA, at high concentration, increased sterility and abortion^38,39^. Regulation of kernel weight by ABA was detected, which was accompanied by an inhibitory effect at high concentration to sucrose synthase and starch synthase activities^40^. In this study, a wheat leaf senescence marker gene, *SAG3*^29^, was upregulated by exogenous ABA treatment and the over-accumulated endogenous ABA in the *tgw1* mutant. Hence, the ABA content change could explain the decrease of chlorophyll contents in the *tgw1* mutant leaves, a crucial component to determine the photosynthesis rate in the source tissue.

In addition, leaf area change could be another negative player for the source energy capture in the *tgw1* mutant. In *tgw1* mutant leaves, the expression of JA-inducible *CNR6* declined, possibly owing to the deficient in JA content. In previous reports, *CNR1* played a negative role in the regulation of organ size and crop yield^41^. In wheat, however, it is very likely that *CNR6* could be a positive regulator on the cell number, which could be tested if necessary genetic materials were available. The reduced cell number and final leaf size played a negative effect on the source for grain filling. Source limitation to GW was noted in some cultivars under different environments^26^. Source for grain filling was found to be a limiting factor when wheat sink capacity increased as exampled in bread wheat recombinant inbred lines with contrasting GN^42^. Therefore, JA defect followed by the accumulation of ABA, a decrease of leaf development, and other unknown factors could all be involved in the GW regulation in *tgw1*.

Following the experimental design on a breeding selection of wheat kernel weight^43^, our field trials showed that *KAT-2B* transgenic lines could significantly increase yield. However, it is noted that the plot size we used (one 1 m row or 1 m^2^ in the 2019 and 2020 experiments, respectively) was smaller than what breeders usually use in breeding selection. In addition, our field trials were conducted in the same location for two years. To validate the breeding applicability of the *KAT-2B* gene, larger-scale field trails across multiple sites with different soil types and environmental conditions are still needed. Furthermore, we used WT rather than null segregant as control. We should point out that null segregant is more appropriate for this purpose.

In conclusion, our work establishes that wheat *KAT-2B* positively regulates GW, a vital component of wheat yield. Consequently, this gene is useful for further dissecting the molecular basis, especially the role of JA, underlying wheat grain development. Because we found that overexpression of *KAT-2B* substantially increased GW and grain yield per plant, the potential value of this gene in developing wheat cultivars with improved GW is worthy of investigation. The use of precise genome engineering techniques, e.g., CRISPR-Cas9^44^, may facilitate the modification of *KAT-2B* function and JA biosynthesis during wheat grain development. Thus, our study offers new clues and possibilities for studying wheat grain development.

## Methods

### Plant materials and growth conditions

All the mutants and transgenic wheat used in this work were in *Triticum turgidum* (tetraploid, AABB genome) Kronos ecotype background. Wild-type seeds were treated with 0.7% EMS for 16 h at 25 °C to generate an EMS mutant library. M_2_ generation siblings were phenotyped to select mutants with precise changes. BC_1_F_2_ generation plants were used for segregation tests, and homozygous plants were selected for subsequent step analyses. *Arabidopsis kat2* mutant was ordered from *Arabidopsis* Research Center (ABRC). All plants were cultivated in a growth chamber at 22 °C, 65% relative humidity, and 16-hour photoperiod. At least two generations of transgenic and EMS mutants were observed with consistent phenotype.

The materials for phenotypic evaluation were grown at the Fudan University Experimental Station (31°27′N, 121°52′E) in Shanghai, representing the southern part of the Yangtze Spring Wheat Zone. For the 2019 experiment, the three genotypes (WT, *tgw1*, *KAT-2B O.E*.) were sown in a single row with 1 m in length spaced 0.25 m apart with 25 plants per row in December 2018, and plants were harvested in June 2019. For the 2020 experiment, each plot consisted of four 1-meter row spaced 0.25 m apart with 25 plants per row and 100 seeds were sown in each plot in December 2019. The four genotypes (WT, *tgw1*, *KAT-2B O.E*. 3^#^, and *KAT-2B O.E*. 4^#^) were arranged using a 4 × 4 lattice design according to a previous publication^43^. Before sowing, compound fertilizer (N: P: K = 25%: 15%: 8%, Hongsifang, Anhui, China) was applied at 191 kg ha^−1^. Field management followed local agricultural practices. In the following June, randomly sampled plants were used to measure tiller number at harvest, total spikelet number per spike, spike length (cm), GNS, GWS, TKW and grain yield per plant. Kernels in the remaining plants were harvested and dried in an oven at 40 °C for a week to a constant weight. Grain yield per square meter was obtained by summarizing the GWs in the plot, and converted to ton per ha for the 2020 experiment.

### BSA to clone *TGW1*

RNA was extracted from mature leaves of 2-month BC_1_F_2_ generation plants using TRIzol Reagent according to the user manual. We next mixed RNA samples from eight individual homozygous plants together at an equal ratio to form one group. We prepared three groups of WT and homozygous mutants, respectively, to construct PE300 libraries and sequenced in Illumina Hiseq4000 (Shanghai Hanyu Bio-Tech). Around 10 Giga high-quality data were collected from each group and analyzed to call homozygous single-nucleotide polymorphisms (SNPs) with at least five reads in any group using the software BCF Tools v0.1.19. Differentially expressed genes were filtered following the rule of |Fold change | > 2, FDR (*q* value) <0.001, and RPKM over 20 in at least one sample using an MA-plot-based method with Random sampling model in DEGseq v1.20.0^45^. Gene assembly and annotation were conducted according to wheat assembly (TGACv1.1).

### Subcellular localization and transgenic plants

*KAT-2B* cDNA was cloned from WT plants, ligated into pDNOR207 by BP reaction (Invitrogen, Life Technologies, Carlsbad, CA, USA), and confirmed by sequencing. For subcellular localization analysis, *KAT-2B* was incorporated into pMDC43 (*Pro*_*35S*_: *GFP-KAT-2B*) by LR reaction (Invitrogen), transformed into *Agrobacterium* GV3101, infiltrated into tobacco leaves, and observed with an inverted confocal microscope equipped with a ×40 oil-corrected objective in Leica TCS SP8 (Leica, Microsystems, Mannheim, Germany) at 48 h^46^. To complement the *Arabidopsis kat2* mutant, *KAT-2* was incorporated into a gateway transgenic vector under the drive of maize ubiquitin (*Pro*_*Ubi*_: *KAT-2-Flag*) by LR reaction, and transformed into *Arabidopsis* by the flower dip method using *Agrobacterium* GV3101. Immature embryos were collected and inoculated with *Agrobacterium* to generate transgenic wheat according to earlier reports^47^.

### Gene expression and protein accumulation analysis

For gene expression pattern analyses, embryos were collected from germinating seeds. Roots were collected from the 1-week seedlings. Stems, shoots, anthers, and pistils were collected from flowering plants. Seven-day-old wheat seedlings were used for various treatments. Seedling leaves were sprayed with 10 μM MeJA, 200 μM DIEC, or 80 μM ABA. Seedings without any treatment were used as controls. RNA was extracted from seedling leaves using TRIzol according to the manufacture’s user manual (Invitrogen). cDNAs were synthesized using the oligo (dT) primer (TAKARA BIO INC, Tokyo, Japan). Quantitative real-time RT-PCR was conducted using SYBR Green Master (TAKARA BIO INC) with gene-specific primers. Primer sequences are provided in Supplementary Table 2.

Total proteins were extracted from leaves of transgenic or WT and analyzed in a Western blot experiment with an anti-KAT-2 antibody generated for this study in a local company (Abmart, Shanghai, China) at 1:400 dilution. A goat anti-mouse IgG HRP (M21001, Abmart) was used to recognize the primary antibody at 1:8000 dilution according to the manufacture user manual. The samples were developed with a basic ECL kit (AB Clonal) in Tanon 5200 Multi imaging system (Tanon Science & Technology Co., Ltd., Shanghai, China). Total protein was extracted from fully expanded leaves in a growth chamber and analyzed as described to check the protein level of complementation lines.

### Recombinant protein purification and activity analyses

Recombinant KAT-2B proteins were purified from Rosetta^48^. *KAT-2B* was incorporated into a gateway expression vector (P_T7_:His9-*AttR:* T_T7_) by LR reaction, and transformed into *E. Coli*, Rosetta, by a heat shock method. For protein induction, 0.5 mM IPTG was added into cell cultures at OD_600_ ~0.8 in a shaker set at 18 °C. Cells were collected from overnight cultures, sonicated to lysis and purified by Ni-NTA beads according to the user manual^49^. For activity assay, 2 μg recombinant KAT-2B proteins were mixed with a reaction buffer with 100 μM CoA and 50 μM acetoacetyl CoA at 25 °C. UV absorbance at 232 nm was read every 0.5 min for 5 min. 100 mM monovalent cations (K^+^ and Na^+^) or 50 mM divalent cations (e.g., Mg^2+^) were added in the reaction buffer to quantify absorbance changes to study the effects of ions on KAT-2B activity. 100 mM phosphate buffers at pH 6, 6.5, 7.0, 7.5, 8.0, 8.5, and 9 were used with 100 mM NaCl to quantify absorbance changes at 25 °C to study the effects of pH on KAT-2B activity.

### Metabolite profiling analysis

Leaves were collected from 1-week-old seedlings and embryos from mature grains. The samples were ground into a fine powder, extracted with 70% ethanol plus 50 μL of internal standard, and analyzed by LC/MS^50^. Quantification of hormones was calculated by comparing with internal isotope standards (JA: C_12_H_13_D_5_O_3_; IAA: C_10_H_7_D_2_NO_2_; ABA: C_15_H_14_D_6_O_4_) dissolved in methanol at 200 ppb^51^ to quantify the contents of plant hormones. For the quantification of endogenous JA content, 2-week old seedling leaves were homogenized in ice-cold phosphate-buffered saline (pH 7.4). After centrifugation at 3000 × *g* for 20 min, the supernatant was assayed by an ELISA kit (Zhen Ke Biological Technology Co., Ltd., Shanghai, China).

Oxidized lipid analysis was performed according to previous literature^52^. In brief, 2 gm of the fully expanded leaf was collected in the field and ground into fine powder in liquid nitrogen in a high-though put homogenizer. In all, 2 mL of cold methanol was added immediately into the sample to kill enzymes. In all, 10 μL of internal standard (2 mg heptadecanoic acid methyl ester/mL hexane) and 50 μL of 1 μM acetic acid was added and mixed well. After centrifugation at 21,000 × *g* for 15 min, the supernatant was removed into a new 10 mL glass tube. Then 1 mL of hexane: ethyl acetate (1:1, v:v) was added and shaken in a 37 °C shaker for 1 hour. The supernatant was removed into the 10 mL tube. After wash three times with hexane: ethyl acetate, 2 mL of ddH_2_O was added and mixed well in the 10 mL glass tube. The solvent phase was removed into a new tube and dried under a flow of nitrogen gas. The sample was dissolved in 0.5 mL of chloroform: isopropanol (2:1, v:v) and loaded into an LC-NH_2_ column. The column was washed twice with chloroform: isopropanol and eluted with ethyl acetate: acetic acid (2:1, v:v). The sample was dried and dissolved in 1 mL of methanol: hexane (4:1, v:v). The sample was cooled in liquid nitrogen for 15 min, mixed with 0.1 mL of acetyl chloride, and kept in the dark overnight. The reaction was stopped with 2.5 mL of 6% K_2_CO_3_ following extraction with hexane. The sample was dried and analyzed by GC/MS. Oxidized lipid characterization was performed based on the mass spectrum, according to a literature^52^.

### Reporting summary

Further information on research design is available in the Nature Research Reporting Summary linked to this article.

## Supplementary information

Supplementary Information

Peer Review

Reporting Summary

Description of Additional Supplementary Files

Supplementary Data 1

Supplementary Data 2

## Data Availability

Data supporting the findings of this work are available within the paper and its Supplementary Information files. A reporting summary for this Article is available as a Supplementary Information file. The data sets and plant materials generated and analyzed during the current study are available from the corresponding author upon request. RNA-seq data have been uploaded to NCBI with accession number PRJNA673987 or wheat expression database in the Triticeae Multi-omics Center [http://202.194.139.32/]. Source data are provided with this paper.

## Source data

Source Data
