# Supplementary material for: Cloning of wheat keto-acyl thiolase 2B reveals a role of jasmonic acid in grain weight determination
Source: Nat Commun. 2020 Dec 8;11:6266. doi: 10.1038/s41467-020-20133-z (PMC7722888; doi:10.1038/s41467-020-20133-z)
Supplement: Supplementary file 1 — Supplementary Information [file 41467_2020_20133_MOESM1_ESM.pdf]

Cloning of wheat *keto-acyl thiolase-2B* reveals a role of jasmonic acid in grain weight determination

Chen and Yan *et al.*

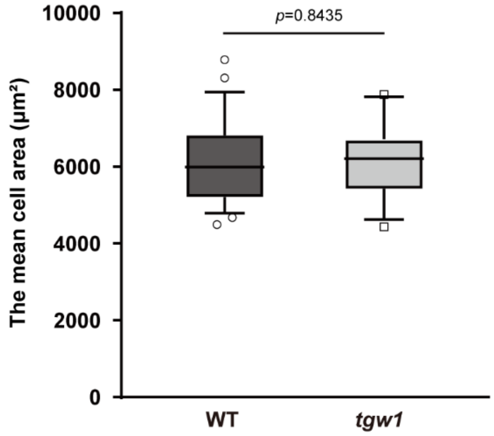

Supplementary Fig. 1. Comparison of epidermal cell areas in leaves from WT (*n* = 53) and the *tgw1* (*n* = 33) mutant. The boxplots indicate median (middle line), 25th, 75th percentile (box) and 5th and 95th percentile (whiskers) as well as outliers (single points). Data are represented as mean ± SEM, and *p*-values are indicated by two-tailed unpaired *t* test. Source data are provided as a Source Data file.

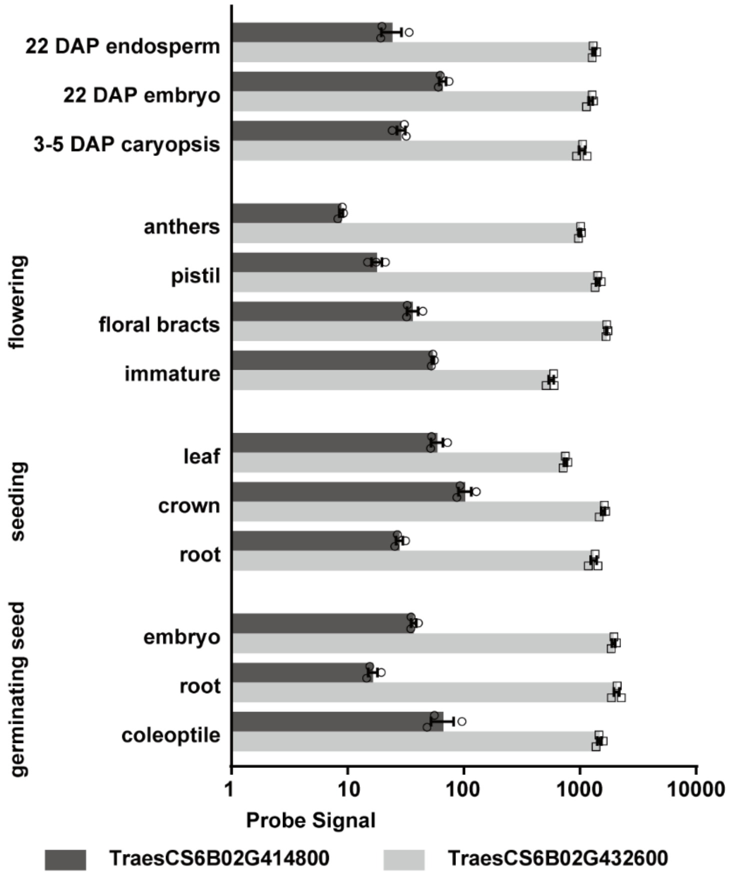

Supplementary Fig. 2. Digital Northern of two candidate genes during wheat development. Data was extracted from GSE12508 in NCBI. n = 3. Data are represented as mean ± SEM. Source data are provided as a Source Data file.

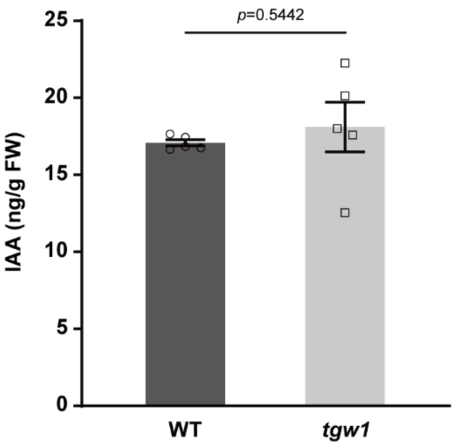

Supplementary Fig. 3. IAA contents in control and the *tgw1* mutant. $n = 5$. Data are represented as mean ± SEM, and p-values are indicated by two-tailed unpaired t test. Source data are provided as a Source Data file.

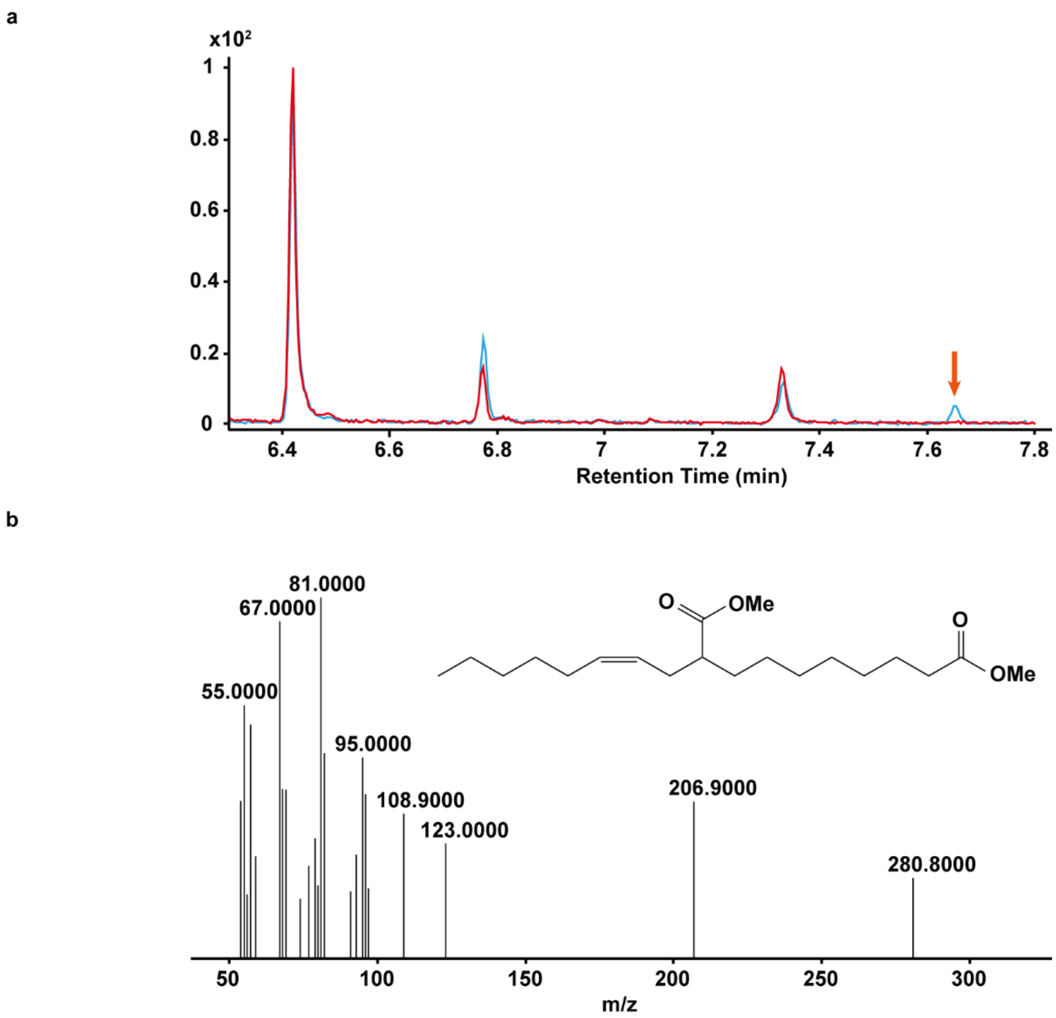

Supplementary Fig. 4. Comparison oxidized lipids in leaves from the *tgw1* mutant and WT. a Chromatograph of oxidized lipid (pointed by the arrow) in leaves of *tgw1* (red) and WT (blue). **b** Mass spectrum of the peak pointed by the arrow in WT. The structure was drawn according to a previous work[1].

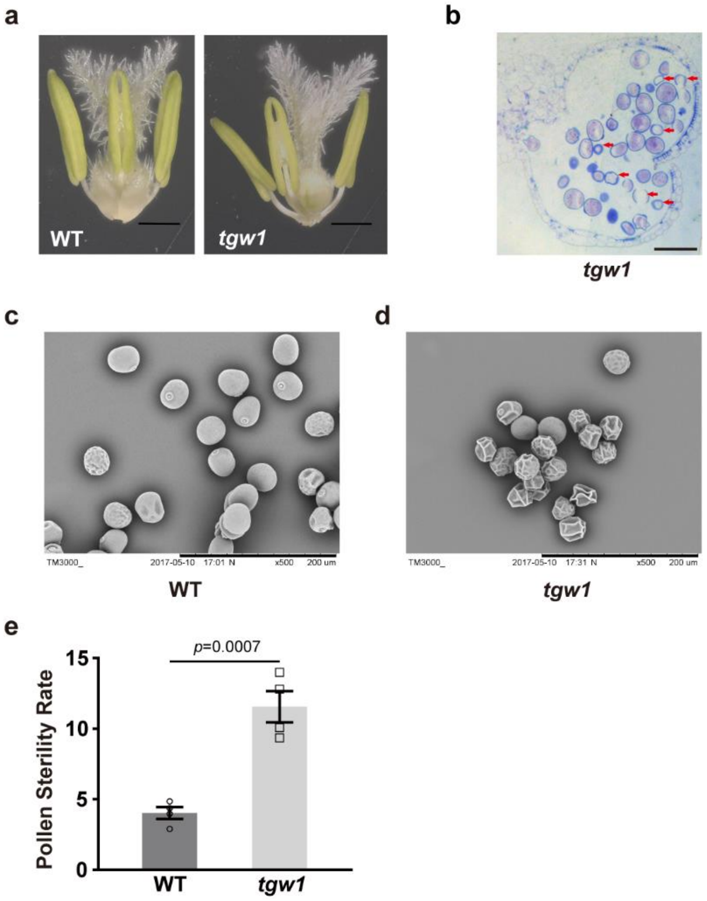

Supplementary Fig. 5. Defect of pollen grains in the *tgw1* mutant. a Anthers were showed from WT or *tgw1* after removing the lemma and palea. Scale bar = 1 mm. **b** Semi-thin cross-section of anthers from *tgw1*. Arrows point to defective pollen grains. Scale bar = 50 μm. **c-d** Scanning electron microscope image to show the morphology of mature pollen grains from WT (c) or *tgw1* mutant (d). Scale bar = 200 μm. **e** Average ratio of defective pollen grains in WT and *tgw1*. $n = 4$. Results in **a-d** are representative of four independent experiments. Data in **e** are represented as mean ± SEM, and *p*-values are indicated by two-tailed unpaired *t* test. Source data are provided as a Source Data file.

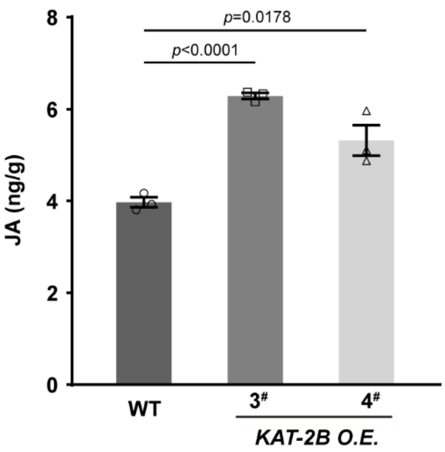

Supplementary Fig. 6. Comparison JA content upon *Fusarium psedograminearum* inoculation at 20 days in *KAT-2B* overexpression lines and WT at the 2-week seedling stage. n = 3. Data are represented as mean ± SEM, and p-values are indicated by two-tailed unpaired t test. Source data are provided as a Source Data file.

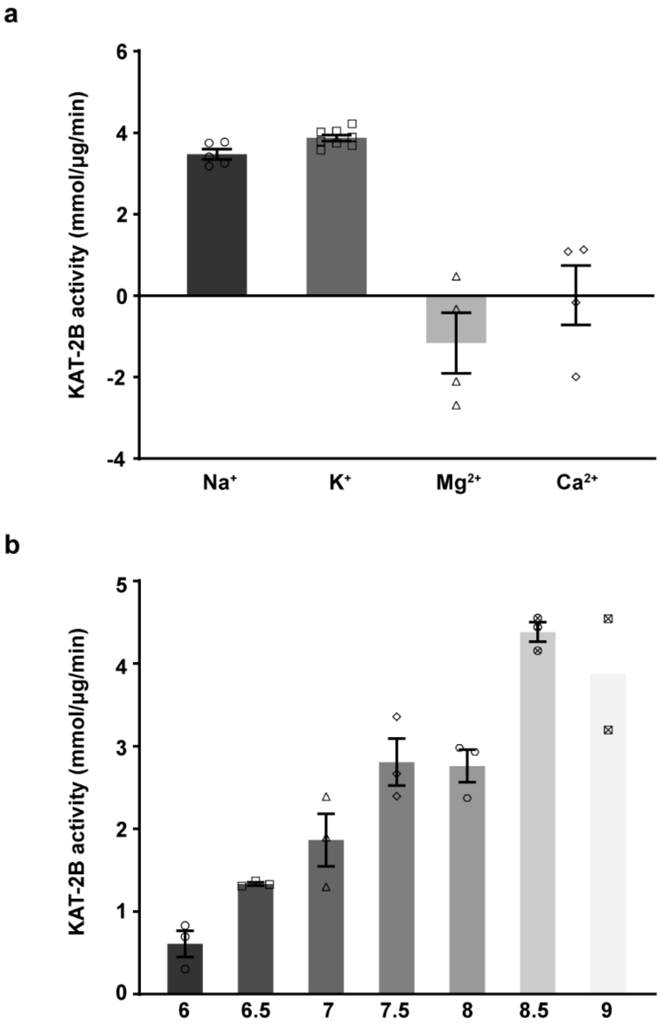

Supplementary Fig. 7. Preferences of recombinant KAT-2B with different ions and pH conditions. a Effect of cation ions on the specific biochemical activity of recombinant KAT-2B. **b** Effect of pH of the reaction buffer on the specific biochemical activity of recombinant KAT-2B. Data are represented as mean ± SEM for at least two independent replicates. Source data are provided as a Source Data file.

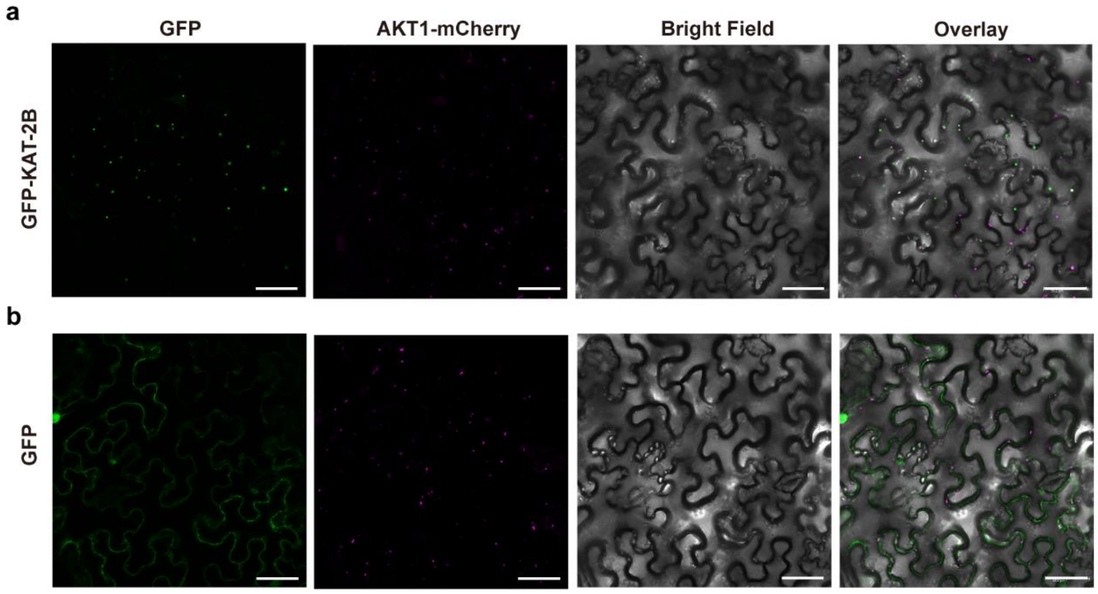

Supplementary Fig. 8. Subcellular localization of GFP-KAT-2B in leaf epidermal cells. **a** Subcellular localization of GFP-KAT-2B fusion protein. **b** Subcellular localization of GFP fusion protein. AKT1-mCherry is a peroxisome marker. Scale bar = 50 μm. Images in **a-b** are representative of two independent experiments. Source data are provided as a Source Data file.

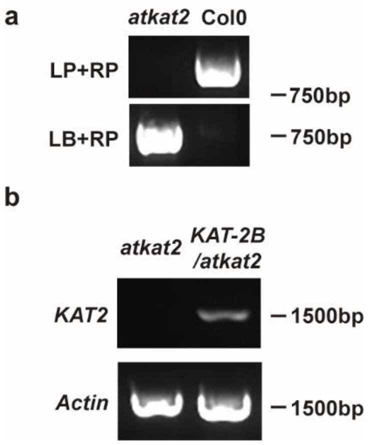

Supplementary Fig. 9. Identification of wheat *KAT-2B* transgenic line in *atkat2* mutant. **a** Genotype validation of *atkat2* mutant. **b** RT PCR analysis of *KAT-2B* in *atkat2* mutant. Images in **a-b** are representative of at least two independent experiments. Source data are provided as a Source Data file.

Supplementary Table 1. Agricultural traits of *tgw1*, overexpression line, and WT control in the field environment in June 2019.

	WT	tgw1	KAT-2B O.E.
TN	15 ± 2.49 $_{n = 10}$	9.67 ± 2.74 $_{n = 12,\ p = 0.0001}$	10.27 ± 1.79 $_{n = 11,\ p < 0.0001}$
SPS	18.91 ± 0.68 $_{n = 54}$	18.07 ± 0.72 $_{n = 55,\ p < 0.0001}$	18.67 ± 0.75 $_{n = 48,\ p = 0.093}$
SPL (cm)	8.68 ± 0.41 $_{n = 55}$	7.17 ± 0.33 $_{n = 48,\ p < 0.0001}$	8.09 ± 0.37 $_{n = 42,\ p < 0.0001}$
GNS	49.24 ± 6.40 $_{n = 46}$	40.55 ± 6.66 $_{n = 40,\ p < 0.0001}$	41.69 ± 5.33 $_{n = 29,\ p < 0.0001}$
GWS	2.77 ± 0.51 $_{n = 30}$	2.25 ± 0.50 $_{n = 37,\ p < 0.0001}$	3.08 ± 0.45 $_{n = 26,\ p = 0.0209}$
TKW (g)	37.96 ± 0.86 $_{n = 7}$	31.80 ± 1.77 $_{n = 5,\ p < 0.0001}$	55.59 ± 1.07 $_{n = 7,\ p < 0.0001}$
GYP (g)	14.25 ± 1.89 $_{n = 6}$	10.32 ± 1.40 $_{n = 8,\ p = 0.001}$	15.95 ± 1.61 $_{n = 11,\ p = 0.0475}$

TN, Tiller number at harvest; SPS, total spikelet number per spike; SPL, Spike length (cm), GNS, Grain number per spike; GWS, Grain weight per spike; TKW, thousand kernel weight; GYP, Grain yield per plant. Data are represented as mean ± SD, and *p*-values are indicated by two-tailed unpaired *t* test. Source data are provided as a Source Data file.

Supplementary Table 2. The primers used for general PCR in this study.

Abbreviation	Primer sequence (5'-3')	Description
KAT-2B-attB1F	GGGGACAAGTTTGTACAAAAAAGCAGGCTTCATGGAGA AGGCGATCGACCG	Construct donor vector
KAT-2B-attB2R	GGGGACCACTTTGTACAAGAAAGCTGGGTTCTTTGCAG CATCCTTGGACAGG	Construct donor vector
KAT-2B-M1F	CTGTAGTTGAGTGATTTGCAGGTGCT	Mutant identification
KAT-2B-M1R	CGACAGTTCATAATTTGCAACAGCAGTG	Mutant identification
KAT-2B-qRT-F	ATGTCGGTGAACTCCGTTG	qRT-PCR primer
KAT-2B-qRT-R	TTGAACTTTCCAGAAGCCG	qRT-PCR primer
Tdβ-Actin -F	ACCTTCAGTTGCCCAGCAAT	qRT-PCR, internal control
Tdβ-Actin -R	CAGAGTCGAGCACAATACCAGTTG	qRT-PCR, internal control
CNR6-F	GGACGAAATGTTCAGGCCCTTAG	qRT-PCR primer
CNR6-R	GCGTTGTTCTCAGCCATGCTCATCTC	qRT-PCR primer
SAG3-F	CGACATCCGACGATTCAAC	qRT-PCR primer
SAG3-R	TCGCACCACCATCCATTC	qRT-PCR primer
PSY-F	CGAGCAGATGGCCGGTCCTG	qRT-PCR primer
PSY-R	ACCCGACATCGGTCCCTTGA	qRT-PCR primer
VDE-F	GATGACTGGTACATTCTCTCATCG	qRT-PCR primer
VDE-R	AACAGCGCCACCATATCCAT	qRT-PCR primer
NCED-F	CGACGAGGTGGTGGTGATCG	qRT-PCR primer
NCED-R	CGGGTGTTGAGGCGGATCTC	qRT-PCR primer
AtActin-F	ATGGCGGACGGTGAAGATAT	RT-PCR, internal control
AtActin-R	GAAGCACTTCCTGTGGACGATC	RT-PCR, internal control
KAT-2-F	ATGGAGAAGGCGATCGAC	RT-PCR primer
KAT-2-R	CTTTGCAGCATCCTTGGACAGG	RT-PCR primer
LBa1	GGTTCACGTAGTGGGCCATC	Mutant identification
LP	TGGGCGCTACAGGTATATCTG	Mutant identification
RP	CTCGACCTCAGGATCACTCAG	Mutant identification

Supplementary Reference

1. Grechkin AN, Ogorodnikova AV, Egorova AM, Mukhitova FK, Ilyina TM, Khairutdinov BI. Allene oxide synthase pathway in cereal roots: detection of novel oxylipin graminoxins. *ChemistryOpen* **7**, 336-343 (2018).
