# Supplementary material for: Cloning of wheat keto-acyl thiolase 2B reveals a role of jasmonic acid in grain weight determination
Source: Nat Commun. 2020 Dec 8;11:6266. doi: 10.1038/s41467-020-20133-z (PMC7722888; doi:10.1038/s41467-020-20133-z)
Supplement: Supplementary file 3 — Reporting Summary [file 41467_2020_20133_MOESM3_ESM.pdf]

nature research

Corresponding author(s): Jin-Ying Gou

Last updated by author(s): Nov 9, 2020

Reporting Summary

Nature Research wishes to improve the reproducibility of the work that we publish. This form provides structure for consistency and transparency in reporting. For further information on Nature Research policies, see our Editorial Policies and the Editorial Policy Checklist.

Statistics

For all statistical analyses, confirm that the following items are present in the figure legend, table legend, main text, or Methods section.

n/a	Confirmed	
[ ]	[x]	The exact sample size (*n*) for each experimental group/condition, given as a discrete number and unit of measurement
[ ]	[x]	A statement on whether measurements were taken from distinct samples or whether the same sample was measured repeatedly
[ ]	[x]	The statistical test(s) used AND whether they are one- or two-sided<br>*Only common tests should be described solely by name; describe more complex techniques in the Methods section.*
[x]	[ ]	A description of all covariates tested
[x]	[ ]	A description of any assumptions or corrections, such as tests of normality and adjustment for multiple comparisons
[ ]	[x]	A full description of the statistical parameters including central tendency (e.g. means) or other basic estimates (e.g. regression coefficient) AND variation (e.g. standard deviation) or associated estimates of uncertainty (e.g. confidence intervals)
[ ]	[x]	For null hypothesis testing, the test statistic (e.g. *F*, *t*, *r*) with confidence intervals, effect sizes, degrees of freedom and *P* value noted<br>*Give P values as exact values whenever suitable.*
[x]	[ ]	For Bayesian analysis, information on the choice of priors and Markov chain Monte Carlo settings
[x]	[ ]	For hierarchical and complex designs, identification of the appropriate level for tests and full reporting of outcomes
[x]	[ ]	Estimates of effect sizes (e.g. Cohen's *d*, Pearson's *r*), indicating how they were calculated

Our web collection on statistics for biologists contains articles on many of the points above.

Software and code

Policy information about availability of computer code

Data collection	BCF Tools v0.1.1.19, Gel Cap, LASAF Application 2.6.7266.0, SkanIt RE for Multiskan GO 3.2, Tanon GEL Capturer, Bio-Rad CFX manager 3.0, nikon D5200, SPAD-502 Plus, HPLC Shim-pack UFLC SHIMADZU CBM20A system, YMJ-D Leaf area measuring instrument, Olympus IX73.
Data analysis	DEGseq v1.20.0, Excel 2019, Agilent MassHunter Workstation Qualitative Analysis B.06.00, TGACv1.1

For manuscripts utilizing custom algorithms or software that are central to the research but not yet described in published literature, software must be made available to editors and reviewers. We strongly encourage code deposition in a community repository (e.g. GitHub). See the Nature Research guidelines for submitting code & software for further information.

Data

Policy information about availability of data

All manuscripts must include a data availability statement. This statement should provide the following information, where applicable:

- Accession codes, unique identifiers, or web links for publicly available datasets
- A list of figures that have associated raw data
- A description of any restrictions on data availability

Data supporting the findings of this work are available within the paper and its Supplementary Information files. A reporting summary for this Article is available as a Supplementary Information file. The datasets and plant materials generated and analyzed during the current study are available from the corresponding author upon request. RNA-seq data has been uploaded to the wheat expression database in the Triticeae Multi-omics Center (http://202.194.139.32/) and PRJNA673987 in NCBI. The source data of this work are provided as a Source Data file.

Field-specific reporting

Please select the one below that is the best fit for your research. If you are not sure, read the appropriate sections before making your selection.

☒ Life sciences ☐ Behavioural & social sciences ☐ Ecological, evolutionary & environmental sciences

For a reference copy of the document with all sections, see nature.com/documents/nr-reporting-summary-flat.pdf

Life sciences study design

All studies must disclose on these points even when the disclosure is negative.

Sample size	No statistical methods were used to predetermine sample sizes. Sample sizes were determined based on previous experience to obtain statistical significance and reproducibility.
Data exclusions	No data were excluded from the analyses.
Replication	All attempts at replication were successful.
Randomization	The field trial were performed a Latin square design.
Blinding	The samples were arranged in a Latin square to exclude effects from soil and growth conditions.

Reporting for specific materials, systems and methods

We require information from authors about some types of materials, experimental systems and methods used in many studies. Here, indicate whether each material, system or method listed is relevant to your study. If you are not sure if a list item applies to your research, read the appropriate section before selecting a response.

Materials & experimental systems

n/a	Involved in the study
☐	☒ Antibodies
☒	☐ Eukaryotic cell lines
☒	☐ Palaeontology and archaeology
☒	☐ Animals and other organisms
☒	☐ Human research participants
☒	☐ Clinical data
☒	☐ Dual use research of concern

Methods

n/a	Involved in the study
☒	☐ ChIP-seq
☒	☐ Flow cytometry
☒	☐ MRI-based neuroimaging

Antibodies

Antibodies used	Anti-Kat2 polyclonal antibody, generated through collaboration with Abmart, Shanghai China, western blot, 1:400<br>Goat Anti-Rabbit IgG-HRP, Abmart, Cat#M21002, western blot, 1:8000
Validation	refer to http://www.ab-mart.com.cn/page.aspx?node=62&id=980 for Goat Anti-Rabbit IgG-HRP.<br>The valence of anti-kat2 was showed in source data file.
