# Supplementary material for: Cloning of wheat keto-acyl thiolase 2B reveals a role of jasmonic acid in grain weight determination
Source: Nat Commun. 2020 Dec 8;11:6266. doi: 10.1038/s41467-020-20133-z (PMC7722888; doi:10.1038/s41467-020-20133-z)
Supplement: Supplementary file 4 — Description of Additional Supplementary Files [file 41467_2020_20133_MOESM4_ESM.pdf]

Description of Additional Supplementary Files

File Name: Supplementary Data 1

Description: Expression levels of *KAT-2A* and *KAT-2B* in 71 tissues of wheat. Data were extracted from an open database (http://bar.utoronto.ca/efp_wheat/cgi-bin/efpWeb.cgi).

File Name: Supplementary Data 2

Description: List of genes differentially expressed between *tgw1* and WT.
