# Supplementary material for: Cloning of wheat keto-acyl thiolase 2B reveals a role of jasmonic acid in grain weight determination
Source: Nat Commun. 2020 Dec 8;11:6266. doi: 10.1038/s41467-020-20133-z (PMC7722888; doi:10.1038/s41467-020-20133-z)
Supplement: Supplementary file 2 — Peer Review [file 41467_2020_20133_MOESM2_ESM.pdf]

Reviewers' comments:

Reviewer #1 (Remarks to the Author):

The authors describe how a premature stop mutation in keto-acyl thiolase 2 (KAT2) is the likely cause of a reduction in grain weight and that JA increases. They speculate on the role of negative regulators of flowering and up regulation of biosynthetic genes. They interpret the results of exogenous ABA applications as a partial mimic/recovery of mutant phenotype. They make the important claim that this work will impact on wheat grain yield and processing quality.
The work is suitable for publication because they have shown that this gene probably functions in wheat as it does in Arabidopsis. However their claim that this work has likely impact on yield and processing quality is incorrect. Although grain size is a numerical component of grain yield it is actually grain number which exhibits sufficient plasticity to deliver large yield increase. The understanding of Fischer et al is not the same as mine. Global wheat yield ranges from less than 1 tonne per hectare to over 15. Grain size hardly differs between these environments it is grain number that changes. Over time, many of the very first wheats produced grains as large, or larger than the highest yielding modern elites. There is no correlation of increased grain size with genetic gain for yield over the decades in the vast majority of environments studied. Grain size, as the authors show, is highly heritable. Breeders place high selection pressure on yield but the component that increases in grain number (low heritability). This is not to say that after maximising grain number, breeders should not then increase grain size as much as they can, while avoiding downwards compensation on grain number. But this ms does not give insights into how that might be done, the authors have show how we can make grains smaller by mutagenesis and return them to normal size by complementation. They have not shown how grain size could be increased. They have not quantified the trade off effects with grain number and they have not measured yield in the field (which is the only place it matters).

Their basic resource is a Kronos EMS population. This and Cadenza hexaploid tilling populations produce dozens of lines with deleterious phenotypes such as the one described here. Do we want to see hundreds of wheat papers describing how you can screw wheat up? Following twenty years of reading papers about how you can screw Arabidopsis up in exactly the same way. I hope not. Authors need to show convincingly how these results are new and, even better, useful.

Specific comments, reviewer in larger font.

12: Yield and processing quality of wheat are determined by grain weight (GW), NO
23: applications to simultaneously improve wheat yield and processing 23

quality.
26: being to beings
26:' is expected' to 'needs to' (we hope it increases this much but on current trends it cannot be expected).
30: The first two dimensions determine grain number (GN) per unit area of land, which has little room to further increase due to GN-GS trade-off as it nearly reaches top limit by excellent efforts of wheat breeders in the past century (Ref 1).
This is not what this paper says and it is certainly not the consensus in the main literature.

54: We build an EMS 55 mutant library using tetraploid spring wheat (Kronos, WT hereafter)
Is this different from the publicly available Kronos EMS population (https://doi.org/10.1073/pnas.1619268114)?

72: sued to used
86: rephrase
155-163: The authors refer to as group of 'flowering related genes'. However, there is no FLC in wheat

and CO does not seem to play any role in flowering regulation in wheat. I suggest the authors rethink this part.

170: The exogenous ABA application seems to be based on quite tenuous evidence. Applying any plant growth regulator to wheat would change grain size if the dose/timing is right. This part, from a harsh critic, could be described as ‘spray and pray’.

180: Collectively, both gene expression and ABA content changes suggested that flowering was delayed in tgw1 mutant while senescence was not.
If the authors want to find out what these phenology phenotypes are, they should measure them directly.

Fig5. There is no FLC in wheat????

Reviewer #2 (Remarks to the Author):

Grain size is important agronomic trait. Chen et al. identified a tetraploid wheat EMS mutant, triticeae grain weight 1 (tgw1), which showed markedly reductions in grain weight. The bulked segregant analysis and genetic transformation indicated that the smaller seed of the mutant phenotype was caused by a premature stop mutation in keto-acyl thiolase 2 (KAT2) involved in β-oxidation during JA synthesis. This result is confirmed and well written. This is the solid new founding of this manuscript. This result is not enough to be published in Nature Communications.
The authors also found that tgw1 mutant delay flowering. And found that several genes in ABA synthesis pathway were up-regulated in tgw1 mutant using the RNASeq data. However, the conclusion that tgw1 mutant activates ABA synthesis to delay flowering is not convincing. Since KAT2 involved in β-oxidation during JA synthesis, it is well known that the reducing of the JA synthesis will impact the expression of many gene. The authors should summarize all the different expression genes between the wild and the mutant first.

Other major concerns:
1. Since the Kronosis is tetraploid spring wheat, are the alleles of the KAT2 the same in A and B chromosomes? Was the expression analysis of the KAT2 carried out using these two alleles ?
2. To assess the potential applications of KAT2 in wheat improvement, it is necessary to compare the agronomic traits in the maturation stage between the mutant and wild type.
3. In fig 1. Only the h has the bar value. It is strange that the defective pollen grains existed in the WT, if the authors can give explain?
4. Which over-expressed transgenic plants was used in the fig3 b,d,e? Why the four KAT2/kat2 plants had different seed weight?

Reviewer #3 (Remarks to the Author):

Compared to dicot plants, much less is known on jasmonate (JA) biosynthesis and its role in monocot plants. Here, the authors describe cloning of a wheat keto-acyl thiolase-2 (KAT2), an enzyme in ß-oxidation of the carboxylic side chain in JA biosynthesis, and its role on grain weight. The story is new, interesting and important for potential improvement of wheat yield and processing quality. Most of the data are convincingly collected. The methods and the experimental design are adequate to the question under study. There are, however, some missing controls, references, and explanations.
In details:

Introduction: The different aspects required for the manuscript are only partially introduced. Whereas grain size (GS) and its positive correlation with grain weight (GW) is briefly mentioned, corresponding QLT´s are summarized in one sentence and linked to plant hormones without details. Even the subsequently presented data of the manuscript require details for JA biosynthesis, there are no comments on KAT2, putative occurrence of KAT gene families in wheat, other monocots or dicots, on the link between mutants in JA biosynthesis and seed development, on comprehensive data for rice etc., please inspect some of the following papers Plant Cell Reports, 2013, 32, 815-827 (review), Interdiscip. Sci, 2015, 7, 7-20 (Oryza, Keto Acyl-CoA synthase), Mol. Cell. Proteomics 2017, 16.11, 1889-1905, (proteomics in wheat and rice), Breed Sci, 2013, 63, 68-71 (AOC in Leymus mollis, wheat), ChemistryOpen 2018, 7, 336-343, (AOS in cereals), Plant Physiol. 2014, 164, 1068-1076, (AOC of wheat).

Results: Starting with an EMS mutant library, a triticeae grain weight (tgw) mutant was isolated and characterized which exhibited reduced size, weight etc. Using a solid genetic strategy, molecular cloning of the underlying gene TGW1 was performed und shown, with help of published microarray data, to be a mutated in KAT2. This enzyme is essential for fatty acid ß-oxidation and biosynthesis of JA and IAA. Indeed, tgw1 seeds showed significant lower JA levels, but fatty acid and IAA levels were not altered, this has to be discussed.

The subsequent validation of TGW1 (KAT2) is well done by complementation analysis, transgenic lines, proof on KAT2 protein, KAT activity of the recombinant protein and phenotypic characterization (Fig. 3. S5). Expression of KAT2 in Arabidopsis kat2 homologous genetic background indicated clearly a rescue of the ß-oxidation defect of the mutant. Subcellular localization of GFP-KAT2 fusion protein was shown for peroxisomes using a peroxisome marker (Fig. 4b). Here, however, better resolution and enlargement via an inset is required. In case of activity tests (Fig. 4c), the legend have to be revised. A change in absorbance, but no change in specific biochemical activity is shown, furthermore, in Fig. 4c a time scale of zero to 5 min is shown, whereas in MM a range of 0.5 min to 20 min is indicated, please revise.

Subsequently, an interesting link of KAT2 and the tgw1 mutant to ABA synthesis/sensitivity and delay in flowering was substantiated by clear data which correspond well to role of ABA in senescence and grain weight/size in other species, further supporting the positive correlation between KAT2 and grain weight.

Beside the phenotypic analysis, biochemical data and expression analysis, the essential detection of JA in tgw1 mutant is done. Here, the method of detection has to be described more carefully, on p. 11, line 296-297 hormone analysis is mentioned according to ref. 40. In ref. 40, however, only quantification of gibberellins are described. Which internal standard and method was used for quantification of JA?

Most of the Discussion is well done, but can be given in more detail. Why there is no altered levels of fatty acids and IAA in the tgw1 mutant? Furthermore, the cross-talk between JA and ABA (cf. Fig. 5 j) can be addressed, there are numerous data on JA versus ABA in cereals. The metabolite profile of oxylipins might be affected in the tgw1 mutant due to the mutation in KAT2; are there graminoxins in tgw1 ?. Please have a look in Grechkin et al., ChemistryOpen, 2018, 7(5) 336-343.

Minor points:
p. 2, line 39, after pathway: write “,” instead of “;”
p. 4, line 89: no proper citation, only ref. 20 fits to the sentence, give original ref.

Reviewers' comments:

Reviewer #1 (Remarks to the Author):

The authors describe how a premature stop mutation in keto-acyl thiolase 2 (KAT2) is the likely cause of a reduction in grain weight and that JA increases. They speculate on the role of negative regulators of flowering and up regulation of biosynthetic genes. They interpret the results of exogenous ABA applications as a partial mimic/recovery of mutant phenotype. They make the important claim that this work will impact on wheat grain yield and processing quality.

The work is suitable for publication because they have shown that this gene probably functions in wheat as it does in Arabidopsis. However their claim that this work has likely impact on yield and processing quality is incorrect. Although grain size is a numerical component of grain yield it is actually grain number which exhibits sufficient plasticity to deliver large yield increase. The understanding of Fischer et al is not the same as mine. Global wheat yield ranges from less than 1 tonne per hectare to over 15. Grain size hardly differs between these environments it is grain number that changes. Over time, many of the very first wheats produced grains as large, or larger than the highest yielding modern elites. There is no correlation of increased grain size with genetic gain for yield over the decades in the vast majority of environments studied. Grain size, as the authors show, is highly heritable. Breeders place high selection pressure on yield but the component that increases in grain number (low heritability). This is not to say that after maximising grain number, breeders should not then increase grain size as much as they can, while avoiding downwards compensation on grain number. But this ms does not give insights into how that might be done, the authors have shown how we can make grains smaller by mutagenesis and return them to normal size by complementation. They have not shown how grain size could be increased. They have not quantified the trade off effects with grain number and they have not measured yield in the field (which is the only place it matters).

Answer: Under field conditions, *KAT2* O.E. plant displayed significant increases in grain weight. *KAT2* O.E. line had reduced grain number per spike and spike number in field conditions, which reflected the GN-GW trade-off. But surprisingly, total grain yield per plant from *KAT2* O.E. plants was 11.9% higher than non-transgenic control.

Based on this data, over-expression of *KAT2* in wheat could increase grain weight (grain size) and produce a potentially positive effect on grain yield per plant in the field condition we tested. Please find this information in Table 1 in the revised version.

These above field data helped a lot to improve our paper quality and also contributed to our investigation of the potential working mechanism! We gratefully appreciate your constructive criticism!

Their basic resource is a Kronos EMS population. This and Cadenza hexaploid tilling populations produce dozens of lines with deleterious phenotypes such as the one described here. Do we want to see hundreds of wheat papers describing how you can screw wheat up? Following twenty years of reading papers about how you can screw Arabidopsis up in exactly the same way. I hope not. Authors need to show convincingly how these results are new and, even better, useful.

Answer: Our data showed that the mutational effects of wheat *KAT2* gene were pleotropic, e.g. negatively affecting JA content and leaf and pollen development, and positively increasing ABA content. In this study, we detected a large amount of differential genes between *tgw1* and WT. The up-regulation of ABA synthesis genes and accumulation of ABA in *tgw1* provided novel nodes

from hormone biosynthesis point of view in the cross-talk network between JA and ABA, which was focused in down-stream signalling pathway before (Plant Cell Rep 32, 815-827).

We agree that mutants with increased grain weight be directly used in breeding programs. The fact that loss-of functional mutation of *KAT2* showed negative effects on grain wheat suggested that the native *KAT2* gene could have a positive effect on grain weight. This positive effect of *KAT2* on GW was supported by the field data.

Field data also showed that the grain yield per plant was increased by 11.9% in the over-expression lines of *KAT2* (Table 1). Therefore, wheat *KAT2* positively regulates wheat yield per plant, possibly through GW. Based on these findings, *KAT2* could be used to further dissect the regulations of GS, GW and GN-GW balance, which may lead to new strategies in raising wheat yield potential.

Please find related data from lines 143 to 154 in the revised version. Thank you!

Specific comments, reviewer in larger font.

12: Yield and processing quality of wheat are determined by grain weight (GW), NO

Answer: We will analyze the processing quality of transgenic plants and report results in future. We removed all information about processing quality in the current text as suggested by the reviewer. Thank you!

23: applications to simultaneously improve wheat yield and processing quality.

Answer: As there is no data to support the processing quality, we removed the word “processing quality” in the text as suggested by the reviewer. Thank you!

26: being to beings

Answer: We changes “being” to “beings” as suggested by the reviewer. Thank you!

26:’ is expected’ to ‘needs to’ (we hope it increases this much but on current trends it cannot be expected).

Answer: We changed “is expected” to “needs to” as suggested by the reviewer. We hope our work could offer new clues and possibilities for raising wheat yield potential. Thank you!

30: The first two dimensions determine grain number (GN) per unit area of land, which has little room to further increase due to GN-GS trade-off as it nearly reaches top limit by excellent efforts of wheat breeders in the past century (Ref 1).

This is not what this paper says and it is certainly not the consensus in the main literature.

Answer: We cited another original paper reporting the GN-GW trade-off. Thank you!

54: We build an EMS mutant library using tetraploid spring wheat (Kronos, WT hereafter)

Is this different from the publicly available Kronos EMS population (https://doi.org/10.1073/pnas.1619268114)?

Answer: There are strict limitations to import seeds into China, which makes it not possible for us to screen the whole publically available Kronos EMS mutant population. Therefore, we treated Kronos seeds in our lab to build a mutant library with over 5,000 independent lines according to the procedure described in the reference (https://doi.org/10.1073/pnas.1619268114).

72: sued to used

Answer: We changed “sued” to “used” according to your suggestion. Thank you!

86: rephrase

Answer: We changed our writing in this sentence. Please find in the revised version. Thank you!
155-163: The authors refer to as group of 'flowering related genes'. However, there is no FLC in wheat and CO does not seem to play any role in flowering regulation in wheat. I suggest the authors rethink this part.
Answer: We only kept the expression of *FRI* gene, which had a strong link with ABA signaling and flowering in early reports, as suggested. Please find this change in Fig. 5f in the revised version. Thank you!
170: The exogenous ABA application seems to be based on quite tenuous evidence. Applying any plant growth regulator to wheat would change grain size if the dose/timing is right. This part, from a harsh critic, could be described as 'spray and pray'.
Answer: The ABA treatment was performed according to a literature about effects of hormones on *Brachypodium* (Sci Rep. 2015 Sep 30;5:14476. doi: 10.1038/srep14476). This concentration (30 μM) was among the range in ABA treatment of maize causing kernel weight reductions (J Plant Physiol. 2017 Sep;216:1-10. doi: 10.1016). Therefore we believe the dose was not an extremely high one.

We agree that other physical changes in *tgw1* mutant could also involve in the regulation of GW and discussed the possibilities in the revised version. Our field data showed that *tgw1* had defected in leave development, which could limit source during grain filling, as reported in early studies (Aust. J. Agric. Res. 29, 16 (1978)). Please find it in the fourth paragraph in the discussion part from line 234 to 248. Thank you!
180: Collectively, both gene expression and ABA content changes suggested that flowering was delayed in tgw1 mutant while senescence was not.
If the authors want to find out what these phenology phenotypes are, they should measure them directly.
Answer: We removed this sentence in the discussion part. Thank you for pointing it out!
Fig5. There is no FLC in wheat????
Answer: In the transcriptome comparison of *tgw1* with WT, FLC was not among the genes with significant changes. Please find the list of differentia genes in the supplemental table. Thank you!

Reviewer #2 (Remarks to the Author):
Grain size is important agronomic trait. Chen et al. identified a tetraploid wheat EMS mutant, triticeae grain weight 1 (tgw1), which showed markedly reductions in grain weight. The bulked segregant analysis and genetic transformation indicated that the smaller seed of the mutant phenotype was caused by a premature stop mutation in keto-acyl thiolase 2 (KAT2) involved in β-oxidation during JA synthesis. This result is confirmed and well written. This is the solid new founding of this manuscript. This result is not enough to be published in Nature Communications. The authors also found that tgw1 mutant delay flowering. And found that several genes in ABA synthesis pathway were up-regulated in tgw1 mutant using the RNASeq data. However, the conclusion that tgw1 mutant activates ABA synthesis to delay flowering is not convincing. Since KAT2 involved in β-oxidation during JA synthesis, it is well known that the reducing of the JA synthesis will impact the expression of many gene. The authors should summarize all the different expression genes between the wild and the mutant first.

Answer: We analyzed differential genes in between *tgw1* and WT. Many genes in the metabolism of several plant hormones (JA, ABA and GA) were detected and listed in Table 2. The suppression of JA metabolism could explain the change of oxidized lipids. The up-regulation of ABA synthesis genes in *tgw1* provided novel nodes in the cross-talk network between JA and ABA, which was focused in down-stream signalling pathway before (Plant Cell Rep 32, 815-827). We also attached the whole list of differential genes in the supplemental information. We hope this information could be useful for scientists in this research field, and our study could offer new clues and possibilities for raising wheat yield potential. Please find them in Table 2 and Table S1 in the revised version. Thank you!

Other major concerns:

1. Since the Kronos is tetraploid spring wheat, are the alleles of the KAT2 the same in A and B chromosomes? Was the expression analysis of the KAT2 carried out using these two alleles?

Answer: We analyzed the expression of *KAT2* by B genome specific primers, and therefore the expression in Fig 3 only represented that in the B genome. However, we did transform the A homeolog into Arabidopsis *kat2* mutant and found it complemented the lethal phenotype in sucrose free medium (Fig. 4d). This data suggested that the A genome homolog of *KAT2* was also functional, which explained the residue JA in *tgw1* mutant.

To evaluate the change of KAT2 in *tgw1* and *KAT2* transgenic plant, we checked the accumulation of KAT2 protein by an anti-KAT2 antibody generated in this study. Fortunately this antibody recognized the KAT2 allele in both A and B genome. The Western Blot data showed that the signal of endogenous KAT2 was much weaker in *tgw1* while those in KAT2 O.E. lines 3 and 4 were stronger than WT. Please find the above information in the Fig. 3c of the revised version. Thank you!

2. To assess the potential applications of KAT2 in wheat improvement, it is necessary to compare the agronomic traits in the maturation stage between the mutant and wild type.

Answer: We collected the agronomic traits in field conditions, as summarized in Table 1. In field conditions, *tgw1* produced grains with lower TGW, but *KAT2* O.E. plant produced grains with higher TGW. These data indicated that the endogenous level of KAT2 protein had a positive correlation with TGW in wheat. Moreover, grain yield per plant was increased in *KAT2 O.E.* plants. The field data suggested that the *KAT2* gene is useful to improve grain weight, and very likely grain yield per plant. This is a very constructive suggestion and greatly helped to improve the quality of our paper! We gratefully appreciate!

3. In fig 1. Only the h has the bar value. It is strange that the defective pollen grains existed in the WT, if the authors can give explain?

Answer: We added bars in Fig1 a, c and d. Please find related information in the revised version. The percentage of viable pollen grains of *P. maritimum* was examined as 81, 4 % using lactophenol-aniline blue staining method (Protoplasma. 2017 Mar;254(2):881-900). Abnormal pollen grains were also reported in Arabidopsis (J Integr Plant Biol. 2017 Sep;59(9):594-599.). Therefore, the existence of defective pollen grain in WT could be normal. Thank you!

4. Which over-expressed transgenic plants was used in the fig3 b,d,e? Why the four KAT2/kat2 plants had different seed weight?

Answer: We used the line 4 in Fig 3 b, d, e, which accumulate more KAT2 protein than WT (Fig 3c). To address the question about different seed weight in KAT2/kat2 lines, we checked the protein level of KAT2 in three lines. In the complemented samples, the KAT2 protein was

accumulated at different levels (Fig. 3h). We believe that the difference in the accumulation of KAT2 protein could very likely contributed to the difference in seed weight in KAT2/kat2 lines. Thank you!

Reviewer #3 (Remarks to the Author):
Compared to dicot plants, much less is known on jasmonate (JA) biosynthesis and its role in monocot plants. Here, the authors describe cloning of a wheat keto-acyl thiolase-2 (KAT2), an enzyme in ß-oxidation of the carboxylic side chain in JA biosynthesis, and its role on grain weight. The story is new, interesting and important for potential improvement of wheat yield and processing quality. Most of the data are convincingly collected. The methods and the experimental design are adequate to the question under study. There are, however, some missing controls, references, and explanations.
In details:
Introduction: The different aspects required for the manuscript are only partially introduced. Whereas grain size (GS) and its positive correlation with grain weight (GW) is briefly mentioned, corresponding QLT´s are summarized in one sentence and linked to plant hormones without details. Even the subsequently presented data of the manuscript require details for JA biosynthesis, there are no comments on KAT2, putative occurrence of KAT gene families in wheat, other monocots or dicots, on the link between mutants in JA biosynthesis and seed development, on comprehensive data for rice etc., please inspect some of the following papers Plant Cell Reports, 2013, 32, 815-827 (review), Interdiscip. Sci, 2015, 7, 7-20 (Oryza, Keto Acyl-CoA synthase), Mol. Cell. Proteomics 2017, 16.11, 1889-1905, (proteomics in wheat and rice), Breed Sci, 2013, 63, 68-71 (AOC in Leymus mollis, wheat), ChemistryOpen 2018, 7, 336-343, (AOS in cereals), Plant Physiol. 2014, 164, 1068-1076, (AOC of wheat).
Answer: In the introduction part, we cited more publications about the metabolism and function of JA in plants. This new paragraph indicated the importance of JA in crop stress resistance and development. Our work here further demonstrated the function of JA in controlling grain weight, and could offer new clues and possibilities for raising wheat yield potential. We appreciate your helpful suggestion!
Results: Starting with an EMS mutant library, a triticeae grain weight (tgw) mutant was isolated and characterized which exhibited reduced size, weight etc. Using a solid genetic strategy, molecular cloning of the underlying gene TGW1 was performed und shown, with help of published microarray data, to be a mutated in KAT2. This enzyme is essential for fatty acid ß-oxidation and biosynthesis of JA and IAA. Indeed, tgw1 seeds showed significant lower JA levels, but fatty acid and IAA levels were not altered, this has to be discussed.
Answer: β-oxidation catalyzes the conversion of IBA to IAA (Plant Physiol 153, 1577-1586 (2010)). In plant, however, IAA was mainly synthesized from l-tryptophan *via* indole-3-pyruvate (IPA) by the TAA and YUC families in Arabidopsis (Biosci Biotechnol Biochem. 2016;80(1):34-42). Our data here agreed with the latter report, and indicated that β-oxidation catalyzed by KAT2 might not play a major role in IAA synthesis in wheat. This could be the reason for the unchanged IAA level in *tgw1* mutant.

JA is one class of oxidized lipids, which primarily occurred in fatty acids esterified in complex lipids. In Arabidopsis, oxidized lipids composition was changed induced upon treatment at 4 □ or pathogen inoculation, but the highest amount upon treatment (~4.3 nmol per mg of dry mass) was around two orders of magnitude lower than those of normal-chain phospholipids and galactolipids (~259 nmol per mg of dry mass) (Plant Physiol. 2012 Jan;158(1):324-39). In a previous study on wheat leaves, contents of oxidized lipids ranged from 0.0005 to 0.015 mass spectral signal per mg dry mass, which was relative very low compared to the total lipids (50 to 200) (Plant Cell Environ. 2016 Mar;39(3):608-17). Therefore, the change of JA biosynthesis could only lead to minimal fluctuation in the content of lipids, which is under detectable in our system.

We discussed potential reasons for changes in fatty acid, IAA and oxidized lipids in the discussion part. Please find them from line 212 to 232 in the discussion part. Thank you!

The subsequent validation of TGW1 (KAT2) is well done by complementation analysis, transgenic lines, proof on KAT2 protein, KAT activity of the recombinant protein and phenotypic characterization (Fig. 3. S5). Expression of KAT2 in Arabidopsis kat2 homologous genetic background indicated clearly a rescue of the ß-oxidation defect of the mutant. Subcellular localization of GFP-KAT2 fusion protein was shown for peroxisomes using a peroxisome marker (Fig. 4b). Here, however, better resolution and enlargement via an inset is required.
Answer: We re-examined the localization of KAT2-GFP in both leaf epidermal cells and protoplasts prepared from mesophyll cells under confocal microscope. Overlapping of KAT2-GFP with AKT1-mCherry (a well-established peroxisome marker) was detected in both experiments with a higher resolution. Please find the related data in Fig 4c and Fig. S6. Thank you!
In case of activity tests (Fig. 4c), the legend have to be revised. A change in absorbance, but no change in specific biochemical activity is shown, furthermore, in Fig. 4c a time scale of zero to 5 min is shown, whereas in MM a range of 0.5 min to 20 min is indicated, please revise.
Answer: We updated the method part for activity assay and re-calculated the biochemical activity based on a standard curve. Please find them in Fig. 4b and Fig. S5. Thank you!

Subsequently, an interesting link of KAT2 and the tgw1 mutant to ABA synthesis/sensitivity and delay in flowering was substantiated by clear data which correspond well to role of ABA in senescence and grain weight/size in other species, further supporting the positive correlation between KAT2 and grain weight.Beside the phenotypic analysis, biochemical data and expression analysis, the essential detection of JA in tgw1 mutant is done. Here, the method of detection has to be described more carefully, on p. 11, line 296-297 hormone analysis is mentioned according to ref. 40. In ref. 40, however, only quantification of gibberellins are described. Which internal standard and method was used for quantification of JA?
Answer: In this experiment, 50 µl of isotope internal standards (JA: $C_{12}H_{13}D_5O_3$; IAA: $C_{10}H_7D_2NO_2$; ABA: $C_{15}H_{14}D_6O_4$) dissolved in methanol at 200 ppb were added into the samples. We added the related information in the method part. Please find it in lines 339 to 340 in page 14 in the revised version. Thank you!

Most of the Discussion is well done, but can be given in more detail. Why there is no altered levels of fatty acids and IAA in the tgw1 mutant? Furthermore, the cross-talk between JA and ABA (cf. Fig. 5 j) can be addressed, there are numerous data on JA versus ABA in cereals. The

metabolite profile of oxylipins might be affected in the tgw1 mutant due to the mutation in KAT2; are there graminoxins in tgw1 ?. Please have a look in Grechkin et al., ChemistryOpen, 2018, 7(5) 336-343.

Answer: We discussed about changes in IAA and fatty acids from line 212 to 232 in page 10 in the discussion part. Based on mass spectrum information, we detected (2′Z)□2□(2□octenyl)□decane□1,10□dioic acid (Product 2 in the reference) in leaves of WT and the *tgw1* mutant according to the method described in the reference (Grechkin et al., ChemistryOpen, 2018, 7(5) 336-343)(Fig. S4). Unexpectedly, Product 2 was declined in *tgw1* mutant, even though it was upstream of beta-oxidation, a theoretically suppressed reaction in *tgw1* mutant. But, this reduction was consistent with the down-regulation of the 14 JA metabolism genes in *tgw1* mutant (Table 2). We discussed this part in the third paragraph in the revised version. Thank you!

Minor points:

p. 2, line 39, after pathway: write "," instead of ";"

Answer: We changed ";" to "," as suggested. Thank you!

p. 4, line 89: no proper citation, only ref. 20 fits to the sentence, give original ref.

Answer: We cited original research papers in this part in the revised version. Thank you!

Reviewers' comments:

Reviewer #1 (Remarks to the Author):

I feel that the impact of this work leans very heavily on the contention that wheat Keto-acyl Thiolase-2 is a 'yield gene'. The authors use the word yield 24 times and round off key sections that this work is going to facilitate yield increases. This is important, as cloning genes in wheat is no big deal nowadays. I am pleased that similar claims for end use quality were removed.

However, the authors still hold on to yield. So you would hope that they presented some yield data. They do not. I do not think it is good enough to talk about 'potential applications to improve yield' for single gene/allele pair candidates- just test it in the field. The authors do report grain 'yield' per plant in the transgenics. That is not yield. Yield is grain weight per unit area, in the field, from plants grown at same density as used in farming.

I think the paper is better placed in a more specialised journal for plant development.

Reviewer #2 (Remarks to the Author):

The authors had answered most of the questions I raised last time. I will only give several minor suggestions about the KAT2 O.E. lines to make the main conclusions more solid.

1. Content of JA in the tgw1 mutant is reduced. Measurement of the JA content in the KAT2 O.E. lines will further support the point that JA promotes grain growth.

2. Similarly, if possible, the content of ABA in the embryos of the KAT2 O.E. lines also should be measured to further clarify the function of KAT2 in ABA synthesis.

3. Fig. 3b and 3d only exhibit one line of KAT2 O.E., more lines should be included to make the conclusion more solid.

4. For Fig. 3g, line 37# forms the largest grains, while the protein level of KAT2 in line37 is not included in Fig. 3h.

5. As shown in Table 1, the spike length (SPL) of KAT2 O.E. is reduced compared with that of WT. The authors should describe and discuss this important result in the manuscript.

Reviewer #3 (Remarks to the Author):

This is a revised version of a previously submitted manuscript on cloning of a Keto-acyl-thiolase2 of rice including its role in jasmonic acid (JA) biosynthesis. The authors prepared carefully the revised version with answers step by step on all concerns of each three reviewers. The revised sentences were highlighted within the manuscript.
Reviewer 1:
Q1: I agree with the answer on KAT-OE lines under field conditions.
Q2: Even here, there were a serious concerns, the authors answered convincingly using the expression data for tgw1 versus WT and highlighting the novel node to ABA.
The remaining answers on specific comments were given completely and can be accepted.
Reviewer 2:
Q1: The authors evaluated as requested the differentially expressed genes much deeper which led to deeper insight in grain yield. That is o.k.

Other major concerns:
1. Again, a convincing answer is given concerning the two alleles of KAT2 by discussing the A and B genome.
2. The answer is o.k.
3. bars were added
4. The protein level was checked, and the answer is o.k.

Reviewer 3:
1. The Introduction was carefully improved including the suggested references
2. Role of ß-oxidation and KAT2 in JA- and IAA- synthesis: the answer is o.k.
3. Localization: the answer and data are o.k. now
4. The legend of Fig. 4 has been revised
5. The answer concerning internal standards is o.k., and the modification within the manuscript are fine
6. Discussion: the requested concern on oxylipin profile in WT versus tgw1 has been satisfactorily addressed and answered.

Reviewer #1 (Remarks to the Author):

I feel that the impact of this work leans very heavily on the contention that wheat Keto-acyl Thiolase-2 is a 'yield gene'. The authors use the word yield 24 times and round off key sections that this work is going to facilitate yield increases. This is important, as cloning genes in wheat is no big deal nowadays. I am pleased that similar claims for end use quality were removed.

However, the authors still hold on to yield. So you would hope that they presented some yield data. They do not. I do not think it is good enough to talk about 'potential applications to improve yield' for single gene/allele pair candidates- just test it in the field. The authors do report grain 'yield' per plant in the transgenics. That is not yield. Yield is grain weight per unit area, in the field, from plants grown at same density as used in farming.

I think the paper is better placed in a more specialised journal for plant development.

Answer: The *tgw1* mutant, two *KAT-2B O.E.* transgenic lines, and wild type control were grown in the field to collect yield data with a Latin square design (see line 315-331 of the revised manuscript for details of the experimental design). The results showed that both *KAT-2B O.E.* transgenic lines had an over 15% increase in the grain weight per unit area. Grain weight and grain yield per plant from the *tgw1* mutant and two *KAT-2B O.E.* transgenic lines repeated the results last year. This yield data supported that *KAT-2B* could serve as a promising gene to improve wheat yield. We feel this result enhances the quality of this work, and thanks for constructive criticize.

Reviewer #2 (Remarks to the Author):

The authors had answered most of the questions I raised last time. I will only give several minor suggestions about the KAT2 O.E. lines to make the main conclusions more solid.

1. Content of JA in the tgw1 mutant is reduced. Measurement of the JA content in the KAT2 O.E. lines will further support the point that JA promotes grain growth.

Answer: We quantified the JA content in two *KAT-2B O.E.* transgenic lines. *KAT2 O.E.-3#* contained significantly more JA than control. *KAT2 O.E.-4#* contained significantly more JA than control upon pathogen infection, although it only had a mild increase without pathogen stress. Interestingly, the yield increase in both lines, suggesting a positive correlation between yield and JA content in the tested range.

2. Similarly, if possible, the content of ABA in the embryos of the KAT2 O.E. lines also should be measured to further clarify the function of KAT2 in ABA synthesis.

Answer: We quantified the ABA content and detected a significant reduction in the *KAT-2B O.E.* transgenic line. This is consistent with the fact the two *KAT-2B O.E.* transgenic lines had higher chlorophyll contents, which was negatively regulated in the *tgw1* mutant with more ABA and early induction of *senescence-associated gene 3*. Please find the results in Fig. 5g-5i, 1j, and 3f.

3. Fig. 3b and 3d only exhibit one line of KAT2 O.E., more lines should be included to make the conclusion more solid.

Answer: We analyzed gene expression, protein accumulation, JA content, and grain yield in two *KAT-2B O.E.* transgenic lines. Please find them in Fig. 3a-3h, and Table 1. Based on these results, a positive correlation occurred between yield and JA content in the tested range.

4. For Fig. 3g, line 37# forms the largest grains, while the protein level of KAT2 in line37 is not included in Fig. 3h.

Answer: We analyzed gene expression, protein accumulation, and grain weight in the four complementation lines. The data showed that line 37# was the one with the highest in gene expression,

the protein content of KAT-2B, and had the most significant increase in grain weight. These data further supported the decisive role of KAT-2B in regulating grain weight. Thank you for the constructive suggestion.

5. As shown in Table 1, the spike length (SPL) of KAT2 O.E. is reduced compared with that of WT. The authors should describe and discuss this important result in the manuscript.
Answer: The new field data did not repeat the change in the spike length, although it repeated the changes in grain weight and numbers. The reduction observed last year could be affected by the growth conditions.

Reviewer #3 (Remarks to the Author):
This is a revised version of a previously submitted manuscript on cloning of a Keto-acyl-thiolase2 of rice including its role in jasmonic acid (JA) biosynthesis. The authors prepared carefully the revised version with answers step by step on all concerns of each three reviewers. The revised sentences were highlighted within the manuscript.
Answer: We gratefully appreciate your positive evaluations! These kindly positive responses encouraged us to continue working on this work. We believe this resubmitted version provided more evidence JA’s role, an essential plant hormone, in regulating the yield of wheat, a critical food crop. Thank you!

Reviewer 1:
Q1: I agree with the answer on KAT-OE lines under field conditions.
Q2: Even here, there were a serious concerns, the authors answered convincingly using the expression data for tgw1 versus WT and highlighting the novel node to ABA.
The remaining answers on specific comments were given completely and can be accepted.
Reviewer 2:
Q1: The authors evaluated as requested the differentially expressed genes much deeper which led to deeper insight in grain yield. That is o.k.
Other majo r concerns:
1. Again, a convincing answer is given concerning the two alleles of KAT2 by discussing the A and B genome.
2. The answer is o.k.
3. bars were added
4. The protein level was checked, and the answer is o.k.
Reviewer 3:
1. The Introduction was carefully improved including the suggested references.
2. Role of ß-oxidation and KAT2 in JA- and IAA- synthesis: the answer is o.k.
3. Localization: the answer and data are o.k. now.
4. The legend of Fig. 4 has been revised.
5. The answer concerning internal standa rds is o.k., and the modification within the manuscript are fine.
6. Discussion: the requested concern on oxylipin profile in WT versus tgw1 has been satisfactorily addressed and answered.

Reviewer #1 (Remarks to the Author):

It is good that the authors have now included some yield data in a well designed replicated trial. Usually a criticism of the experimental design would be the use of 1m2 plots eg no wheat breeder would trust yield data from such small plots. However, if I have understood correctly, the reported yield increase is now very large indeed. In wild type it is just under 4 tonnes per hectare and in the two over expression transgenics 5-5.2 tonnes, a 20% increase over Kronos (wt). That is an unprecedented single gene increase in wheat yield even when compared to the Green Revolution gene Rht-1. So it is very important that these exciting results stand up to post publication scrutiny. As far as I can see, the t test was used to compare Kronos to the two overexpression transgenics. It is standard practice when assessing transgenic lines to compare the phenotype of null segregants with lines carrying the transgene. I assume that this is a requirement of Nature Communications. As I say a 20% yield increase, especially one based on grain size, is unprecedented so it is really important to check this. There might be a technical reason why the authors were not able to make the comparison with null segregants. Can they expand on this point.

Reviewer #2 (Remarks to the Author):

The authors had answered the questions I raised last time. I think this manuscript can be accepted now.

Reviewer #1 (Remarks to the Author):

It is good that the authors have now included some yield data in a well designed replicated trial.
a criticism of the experimental design would be the use of 1m2 plots eg no wheat breeder would trust yield data from such small plots. However, if I have understood correctly, the reported yield increase is now very large indeed. In wild type it is just under 4 tonnes per hectare and in the two over expression transgenics 5-5.2 tonnes, a 20% increase over Kronos (wt). That is an unprecedented single gene in wheat yield even when compared to the Green Revolution gene Rht-1. So it is very important that these exciting results stand up to post publication scrutiny. As far as I can see, the t test was used to compare Kronos to the two overexpression transgenics. It is standard practice when assessing lines to compare the phenotype of null segregants with lines carrying the transgene. I assume that this a requirement of Nature Communications. As I say a
20% yield increase, especially one based on grain size, is unprecedented so it is really important to this. There might be a technical reason why the authors were not able to make the comparison with null segregants. Can they expand on this point.

Answer: In the discussion part we added "*KAT-2B* transgenic lines increased yield significantly in our field trial experiment following recent work on a breeding selection of kernel weight[44]. However, the plot size we used here (1 m^2) are smaller than breeders usually use in breeding practice. WT plant is a reasonable control for the *tgw1* mutant, but null segregant with lines carrying the *KAT-2B* transgene could be more appropriate to compare the transgenic lines. Moreover, multiple sites with different soil types and environmental conditions in larger scale are still needed to test the gene's applicability in real agricultural practice."

Reviewer #2 (Remarks to the Author):

The authors had answered the questions I raised last time. I think this manuscript can be accepted now.
Answer: Thank you!
